# Incorporating uncertainty in Indigenous sea Country monitoring with Bayesian statistics: Towards more informed decision-making

**DOI:** 10.1007/s13280-024-01980-2

**Published:** 2024-02-14

**Authors:** Katherine Cure, Diego R. Barneche, Martial Depczynski, Rebecca Fisher, David J. Warne, James McGree, Jim Underwood, Frank Weisenberger, Elizabeth Evans-Illidge, Brendan Ford, Daniel Oades, Azton Howard, Phillip McCarthy, Damon Pyke, Zac Edgar, Rodney Maher, Trevor Sampi, Kevin Dougal, Bardi Jawi Traditional Owners

**Affiliations:** 1 Australian Institute of Marine Science, Indian Ocean Marine Research Centre, The University of Western Australia (MO96), Entrance 4, Fairway, Crawley, WA 6009 Australia; 2 UWA Oceans Institute and School of Biological Sciences, The University of Western Australia, Crawley, WA 6009 Australia; 3 School of Mathematical Sciences, Faculty of Science, Queensland University of Technology, 2 George Street, Brisbane, QLD 4000 Australia; 4 Centre for Data Science, Queensland University of Technology, 2 George Street, Brisbane, QLD 4000 Australia; 5 Gondwana Link Ltd, 70-74 Frederick St, PO Box 5276, Albany, WA 6332 Australia; 6 Frank Weisenberger Consulting, 13A Jessie Street, Coburg, VIC 3058 Australia; 7 Kimberley Land Council, 11 Gregory St, Broome, WA 6725 Australia; 8 Bardi Jawi Rangers, Kimberley Land Council, Bardi Jawi Rangers Office, Lot 19-20 First Street, One Arm Point, Ardyaloon, WA 6725 Australia; 9 Australian Institute of Marine Science, 1526 Cape Cleveland Road, Cape Cleveland, QLD 4810 Australia

**Keywords:** Baited Remote Underwater Video Stations, Bayesian multilevel models, Fisheries management, Healthy Country Plans, Indigenous monitoring, Marine Protected Areas

## Abstract

Partnerships in marine monitoring combining Traditional Ecological Knowledge and western science are developing globally to improve our understanding of temporal changes in ecological communities that better inform coastal management practices. A fuller communication between scientists and Indigenous partners about the limitations of monitoring results to identify change is essential to the impact of monitoring datasets on decision-making. Here we present a 5-year co-developed case study from a fish monitoring partnership in northwest Australia showing how uncertainty estimated by Bayesian models can be incorporated into monitoring management indicators. Our simulation approach revealed there was high uncertainty in detecting immediate change over the following monitoring year when translated to health performance indicators. Incorporating credibility estimates into health assessments added substantial information to monitoring trends, provided a deeper understanding of monitoring limitations and highlighted the importance of carefully selecting the way we evaluate management performance indicators.

**Supplementary Information:**

The online version contains supplementary material available at 10.1007/s13280-024-01980-2.

## Introduction

Recognition of the profound cultural and spiritual connections of First Nations peoples with their environment and the benefits of their inclusion in adaptive ocean management is building momentum worldwide (Lauer and Aswani 2010; Artelle et al. 2019; Houde et al. 2022). A millennial history of marine stewardship using traditional ways has maintained healthy ecosystems in sea Country—the Indigenous concept of traditional coastal and sea estates and their associated set of cultural, spiritual, and environmental values (Rist et al. 2019; Fischer et al. 2022)—across remote tropical Australia. Despite this history, Indigenous peoples’ inclusion in government-led ocean management has been mostly limited, partly because the conservation and management landscape is biased to western science and governance structures that are foreign to Indigenous cultures (Ross et al. 2009; Peer et al. 2022). Practices are often limited to tick-box-type exercises of stakeholder engagement (Strand et al. 2022), with limited opportunity to genuinely influence or co-design the decision-making process (Smit et al. 2022). The United Nations Declaration on the Rights of Indigenous Peoples established a universal framework to uphold the rights and interests of Indigenous people including their role in management and governance of land and sea Country. Governments and researchers are increasingly creating spaces for Indigenous roles and perspectives to improve our collective understanding and management of natural resources (Nakashima et al. 2018; Souther et al. 2023). Key to the success of these efforts are genuine two-way partnerships between Indigenous peoples and science organisations that move beyond knowledge inequities to fully integrate traditional and contemporary knowledge in a way that equally shares their power and participation (Hill et al. 2012).

Australia’s first peoples, the Aboriginal and Torres Strait Islander Peoples, represent the oldest continuous culture on Earth (Malaspinas et al. 2016). For over 50,000 years they have established deep spiritual and cultural connections to Country of the Australian continent and adjacent seas. As Traditional Owners (TOs) and custodians of Australia’s land and sea Country, their rights and interests include the cultural responsibility to look after Country and safeguard it for future generations (Rist et al. 2019). This has resulted in vast holdings of traditional knowledge based on detailed observation and experimentation, transmitted between generations through cultural expressions and traditions, encompassing climate shifts and major sea level changes to coastline and island systems (Horstman and Wightman 2001; Nunn and Reid 2016). With this immense body of knowledge, Indigenous Australians have managed Australia’s marine ecosystems for tens of thousands of years (Allen and O’Connell 2003; Nunn and Reid 2016). Currently however, even the most remote marine ecosystems are under increasing pressure from climate change, habitat loss, fisheries, and tourism (Wilson et al. 2006; Graham et al. 2008; Wilson et al. 2012; Jones et al. 2018). These new challenges require cooperation between Indigenous peoples, research organisations, and government agencies, to improve understanding of marine and coastal ecosystems and improve management and social outcomes (Ross et al. 2009; Dobbs et al. 2016; Rist et al. 2019).

The role of Indigenous peoples in managing land and sea Country in Australia and actively participating in its governance, has been re-asserted in the past three decades by a combination of joint management arrangements in marine protected areas (MPAs), designation of Indigenous Protected Areas (IPAs), and funding of over 170 community based Indigenous Ranger groups employing over 1900 rangers to support conservation (Ross et al. 2009; Rist et al. 2019). Indigenous Protected Areas have been established in Indigenous-owned land or sea and currently make up about 50% of Australia’s National Reserve System (Rist et al. 2019). They empower TOs with official recognition and resources for governance, ranger employment, and operational funds. These forms of protected areas are established through a formal agreement with government to promote conservation of biodiversity and cultural resources; they are accompanied by the development of a management plan which sets out how TOs propose to look after land and sea Country. Since the first Healthy Country Plan was developed by the Wunambal Gaambera people in the Northern Kimberley, many other TO groups in Australia chose the Healthy Country Planning methodology, an adaptation of the widely used Open Standards for the Practice of Conservation, to develop IPA management plans (Conservation Measures Partnership 2020). Healthy Country Plans outline key targets for conservation and set out strategies to abate threats, restore targets, and evaluate their ongoing health and impacts through performance indicators. Science partnerships are essential elements of management plans in IPAs; they can provide training and assist in the collection of systematic monitoring data to inform, implement and evaluate management actions (Depczynski et al. 2019; Rist et al. 2019; Souther et al. 2023).

The Australian Institute of Marine Science (AIMS) is a nationally sponsored scientific research agency tasked with conducting marine monitoring as one of its core priorities. Since 2018, AIMS has been working in partnership with Indigenous communities across northern Australia to co-design monitoring programs and inform sea Country management. Within this program, AIMS has partnered with the Bardi Jawi Rangers in the remote Kimberley region of northwest Australia to monitor coral reefs and fish populations in the Bardi Jawi IPA. Using modern science and technologies underpinned by Traditional Ecological Knowledge, the partnership has co-designed a cross-cultural monitoring program that targets key indicators in their management plan (Bardi Jawi Niimidiman Aborginal Corporation 2012; Depczynski et al. 2019) to inform marine management strategies.

Monitoring is the repeated sampling over space and time to collect baseline data and evaluate spatial and temporal variation so that reliable estimates of change can be obtained to inform reactive management decisions (Magurran et al. 2010; Nash and Graham 2016). Monitoring provides information on the impact of pressures to marine environments and can identify trends and vulnerabilities. Ideally, this information should be translated into improved decision-making to avoid the degradation of cultural, ecological, and social values (Emslie et al. 2008; Babcock et al. 2010). To be impactful, particularly in Indigenous-partnered science and marine management contexts, monitoring trends need to be effectively communicated between scientists, TOs, community, and decision makers—this is a particularly challenging task because of the different streams of knowledge (Strand et al. 2022). Moreover, statistical trends carry uncertainty in their estimates depending on the monitoring spatiotemporal design and analysis models. Such uncertainty needs to be carefully reported on and considered to provide transparent pathways to management decision-making, and to demonstrate a realistic evaluation of western science to sensitively detect changes for TOs.

Bayesian statistics offer a powerful way to model spatiotemporal trends and their uncertainty. Bayesian inference combines prior information with information gained from the data, to yield posterior distributions for the model and its parameters; parameter probabilities and their uncertainty can then be directly calculated from these distributions (Kruschke and Liddell 2018). For fish populations, which exhibit high levels of abundance variation in space and time (Holbrook et al. 1994; Anderson and Millar 2004; Cure et al. 2018), providing probabilistic information on population trends is essential to inform more rapid and confident decision-making in an adaptive management setting. Consider, for example, a scenario in which changes in fish abundance over time are used to categorise the health status of a population based on pre-determined health categories. In Bayesian statistics, the posterior distribution of fish abundance yields not only the central estimate (e.g., changes in mean or median) but also the probability (a.k.a. credibility) of that abundance estimate encompassing any predetermined health status category. Therefore, by calculating probabilities for selection of each possible health category for an indicator, Bayesian models can provide a direct and interpretable measurement of the uncertainty and credibility of detected changes between sampling events. In most marine monitoring programs, this credibility is expressed in a qualitative way, mostly informed by the opinion of scientists and, in some cases, Traditional Owners (McField and Kramer 2017; Trebilco et al. 2021; Great Barrier Reef Marine Park Authority 2022).

Here, we present an inter-disciplinary case study combining knowledge systems of Indigenous Rangers, community members, non-Indigenous ecologists and statisticians, to design and evaluate results from a monitoring plan for fish populations of traditional and recreational importance in coral reef habitats within the Bardi Jawi IPA. We specifically showcase how using Bayesian multilevel models to assess changes through time in fish abundance indicators, can contribute degree of credibility information that directly aligns with the traffic light system used in Healthy Country Plans and many worldwide monitoring programs to assess ecosystem health (Bardi Jawi Niimidiman Aboriginal Corporation 2012; Bardi Jawi Rangers 2020). We suggest that incorporating this uncertainty information has the potential to improve the reporting of monitoring trends and potentially increase the impact of monitoring data on management outcomes, by allowing sea Country managers to better understand the true state of the environment and the natural variations associated with fish abundance data. We conclude our work by discussing how we envision this approach evolving further and in a manner that can be adopted and adapted by coastal Indigenous communities and their partners across Australia and the globe.

## Materials and methods

### Monitoring co-design

**Fig. 1:**
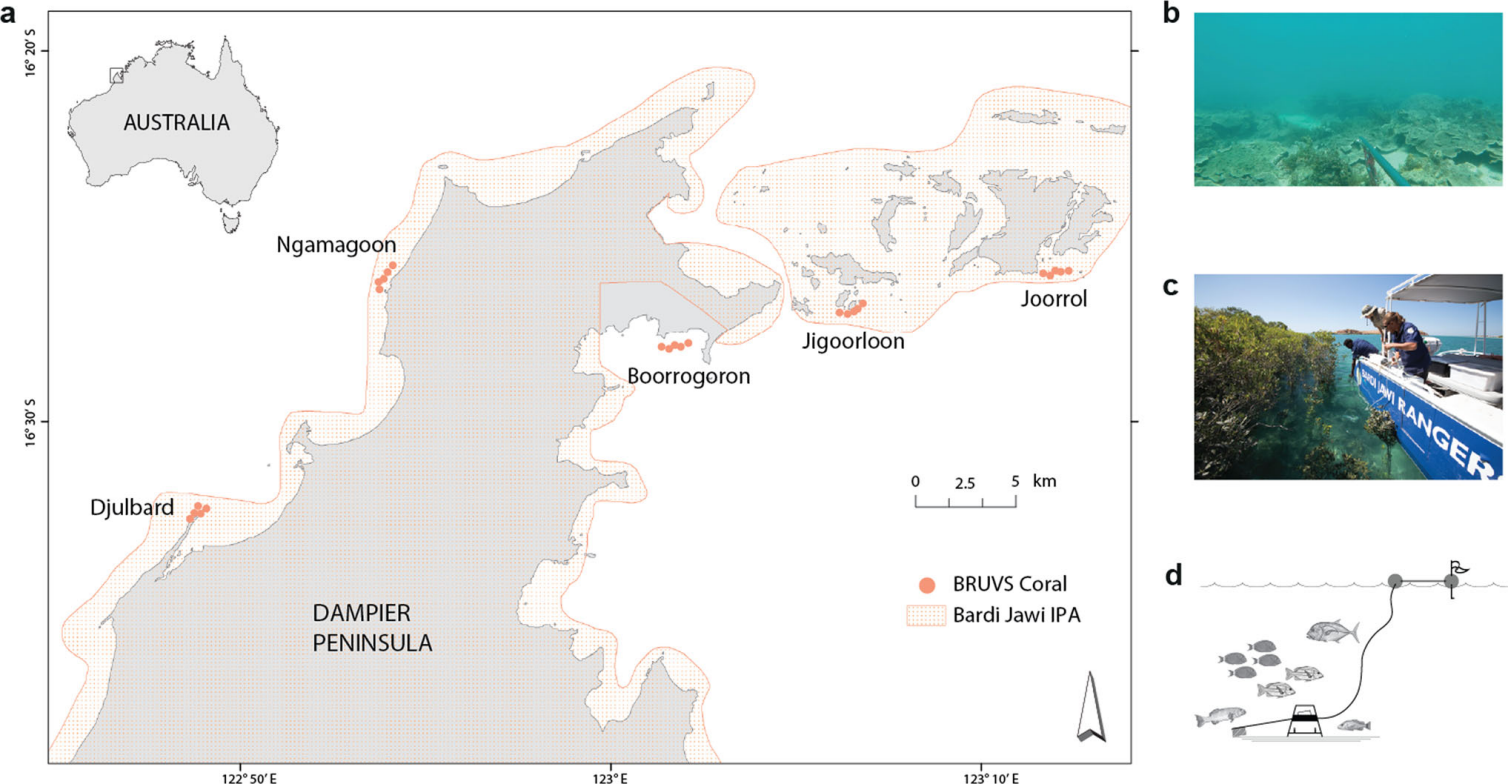
Map of the Bardi Jawi IPA monitoring sampling locations for *aarli* (fish) in coral reef habitat (**a**). Side panel shows an example of coral reef habitat (**b**), as well as the Bardi Jawi Rangers and AIMS staff deploying Baited Remote Underwater Video Stations (BRUVS) (**c**), and a schematic diagram of these video stations in the seabed (**d**)

The Bardi Jawi Native Title Determination includes over 204,000 hectares of sea Country and 200 km of coastline along the Dampier Peninsula in northwest Australia (Fig. 1a). An IPA was established in 2013, with the vison of maintaining healthy land and sea Country, as well as traditional cultural knowledge and practice for future generations (Bardi Jawi Niimidiman Aborginal Corporation 2012). Under the direction of their Elders, the Bardi Jawi Rangers, who are also Traditional Owners of Bardi Jawi Country and include representatives across clan groups, are responsible for implementing the IPA Management Plan, monitoring the health of its key conservation targets, and reporting outcomes back to the community. Overlapping the Bardi Jawi IPA is the Bardi Jawi Gaarra Marine Park, a newly appointed joint management marine park that has been co-designed between state government and TOs, including the Bardi Jawi Rangers.

Bardi Jawi sea Country is rich in biodiversity, with high levels of endemism and a mosaic of habitat types, including inter-tidal pools, mangroves, seagrass, algal beds, and well-developed coral reef systems (Fox and Beckley 2005; Thorburn et al. 2007). Tidal fluctuations in this region are one of the largest in the world, reaching up to 12 m and creating tidal currents of up to 10 knots (Purcell 2002; Lowe et al. 2015). Bardi Jawi people are saltwater people, custodians of their sea Country and have historically deep and strong biophysical knowledge and connections to the sea, on which they depend for their livelihood and food security. In particular, fish (*aarli* in Bardi Jawi language) are an abundant and readily accessible resource for the Bardi Jawi people.

In 2018, AIMS was invited by the Bardi Jawi Rangers as a partner to co-design a marine monitoring program for the Bardi Jawi IPA. Merging the expertise of scientists at AIMS in coral reef and fish monitoring, the traditional knowledge held by rangers and Elders, and key conservation targets in their IPA Management Plan (*marnany*—coral reef, and *aarli*—fish), we co-designed a monitoring program during an on-Country workshop (see Depczynski et al. 2019). We used a participatory mapping approach (Davies et al. 2020) to collect Traditional Ecological Knowledge on local habitats and species, and establish habitat maps for the area. We then used this knowledge and maps to select monitoring sites and design a monitoring program that considered ranger working capacity, included sites within different clan areas, accounted for different levels of human access, and targeted areas of known habitat of culturally important species and resources (Depczynski et al. 2019). During this workshop, AIMS also provided training to rangers about quantitative scientific monitoring, sampling techniques and technologies, with an emphasis on the use of long-term monitoring data as a tool for detecting change, reporting to government and management agencies, and participating in policy change. Despite this work being fully co-designed, we discussed the need for Free Prior and Informed Consent of the Traditional Owner community and co-developed a data sovereignty agreement between organisations to protect Bardi Jawi data, knowledge and participation, following guidelines in the AIATSIS Code of Ethics for Aboriginal Research (Australian Institute of Aboriginal and Torres Strait Islander Studies 2020).

### Sampling fish populations

To sample fish populations (*aarli*) in Bardi Jawi sea Country, we used Baited Remote Underwater Video Stations (BRUVS)—henceforth video stations, a non-destructive western science developed diver-less method (Cappo et al. 2003; Whitmarsh et al. 2017). This method alleviates the need for expert fish identification in the field, ensures a long-term record of the fish community at each location and is also the safest option to sample fish populations in the region because of its high-energy tidal currents and the abundance of sharks and crocodiles.

Video stations were deployed annually from 2018 to 2022, during August–September, targeting slack water at low tide during neap tides and a window of most favourable wind and swell conditions. Because environmental conditions vary drastically along the coastal Kimberley region, selection of consistent deployment times is crucial for minimising variation in currents and visibility, which could influence fish counts. All samples were taken in full daylight to avoid the effects of crepuscular times in fish behaviour (Helfman 1986), despite some variation in time of day being inevitable given tide selection is prioritised and tide times vary considerably from year to year. All deployments were at coral reef habitats, which in the Kimberley, are characterised by patchy coral communities of low coral cover (~ 5 to 20%), interspersed with macroalgae and soft corals. Minor coral bleaching was observed in 2016 (Gilmour et al. 2019) prior to the beginning of this work for the first time in the region, as documented by hundreds of years of oral history, and again in 2019. Bleaching was not observed during the rest of the study period; therefore, major bleaching effects that could affect reef structure and have a large-scale influence on fish populations were assumed to be minor.

Samples were collected in shallow water (5–7 m at low tide) at five sites spanning the western and eastern sides of the Dampier Peninsula. Site selection combined traditional knowledge and western science to include different clan areas, account for different levels of human access, and be representative of coral reef habitat (> 5% coral cover). Moreover, all sites remained submerged even during lowest tides (Fig. 1a; Table S1). One site, Boorrogoron, was only sampled twice (2020 and 2022) due to logistical constraints. Five 30-min deployments, separated by a minimum distance of 250 m, were randomly undertaken at each site for a total of 20 video samples each year (25 samples in 2020 and 2022, when Boorrogoron was surveyed). Before initial deployment, habitat was visually assessed using an underwater viewer to ensure we were targeting coral reef habitats consistently. Annual sampling then targeted the same coordinates for video stations to minimise spatial variation over time. Cameras (GoPro Hero5 Black, 30 frames per second, 1920 × 1080-pixel resolution) were placed on a lightweight stainless-steel frame separated by 380 mm and faced a bait bag filled with 1 kg of crushed pilchards (*Sardinops sagax*).

Imagery from video stations was analysed using EventMeasure software (www.seagis.com.au) to determine fish species diversity and abundance as *MaxN*, a relative measure of abundance (maximum number of individuals from each species viewed at a single still frame during each video sample; Ellis and DeMartini 1995; Willis and Babcock 2000). All fish in the videos were identified to the lowest taxonomic level possible and treated as species complexes in cases where identification based on video imagery was not possible (i.e., *Plectropomus* spp., coral trout complex). *MaxN* estimates were then extracted for each of the ten species or groups of species selected as important indicators by the Bardi Jawi Rangers, including Elders with senior cultural authority, for their importance in traditional and recreational fisheries (Table 1). Data presented in this case study focuses on this subset of the fish community. For each video sample, we also estimated current speed and water clarity using an ordinal scale of 0–5 and predicted tidal amplitude at time of deployment in metres (see Appendix S1 for details).

**Table 1:** Ten *aarli* (fish) species or group of species identified as important indicators of the health of populations important for food security in Bardi Jawi sea Country

Bardi Jawi name	Common name	Species or Group
Barrambarr	Bluebone	*Choerodon schoenleinii*
Barrbal	Rabbitfish	*Siganus lineatus*
Biidib	Rock cods	*Epinephelus* spp.
Biindarral	Coral trout	*Plectropomus* spp.
Gambarl	Surgeonfish	*Acanthurus grammoptilus*
Goolan	Bluespot tuskfish	*Choerodon cyanodus*
Irrariny	Grass Emperor	*Lethrinus laticaudis*
Jirral	Trevallies	*Carangoides* spp.*, Caranx* spp.*, Gnathanodon* spp.
Jooloo	Stripey Snapper	*Lutjanus carponotatus*
Maarrarn	Mangrove Jack	*Lutjanus argentimaculatus*

This list includes species important to both Indigenous and recreational fisheries

### Healthy Country Plan

Fish (*aarli*) are one of seven culturally important targets within the Bardi Jawi IPA Management Plan with a goal of restoring health to these targets identified by TOs as the most important to be looked after (Bardi Jawi Niimidiman Aboriginal Corporation 2012). Bardi Jawi people have concerns that fish are threatened by increased recreational fishing, especially following improved road access to their sea Country since 2019, and want to make sure that this food source continues to be available now and for future generations.

**Fig. 2:**
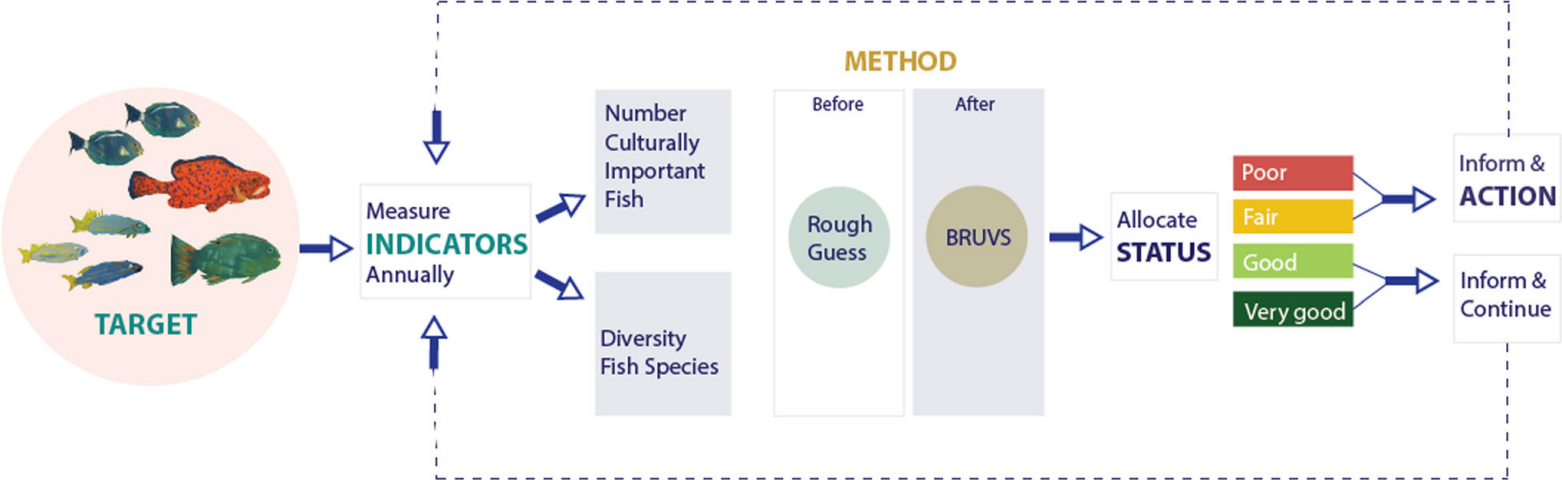
Diagram detailing the process for evaluation of management targets as part of the Bardi Jawi IPA Management Plan. Here we show the process for evaluating the health of *aarli* (fish) based on two indicators, which are currently evaluated via video stations, following partnership between rangers and scientists. Fish in this image were painted by Bardi Jawi children during an on-Country workshop to share monitoring results with community

Through their healthy Country planning, Bardi Jawi people have also developed a plan to monitor the outcomes from implementing their IPA plan (Bardi Jawi Rangers 2020). This plan includes a set of indicators used to monitor the health of management targets and document their status based on a traffic light system, similar to many monitoring programs around the world. Categories for this system are: red—*poor*—restoration is very difficult and may result in extinction, yellow—*fair*—outside acceptable range of variation and requires human intervention, light green—*good*—within acceptable range of variation and some intervention required for maintenance, and dark green—*very good*—most desirable status and requires little intervention for maintenance. Adjustments to management are then made based on these assessments to restore indicator health where needed (Fig. 2).

**Fig. 3:**
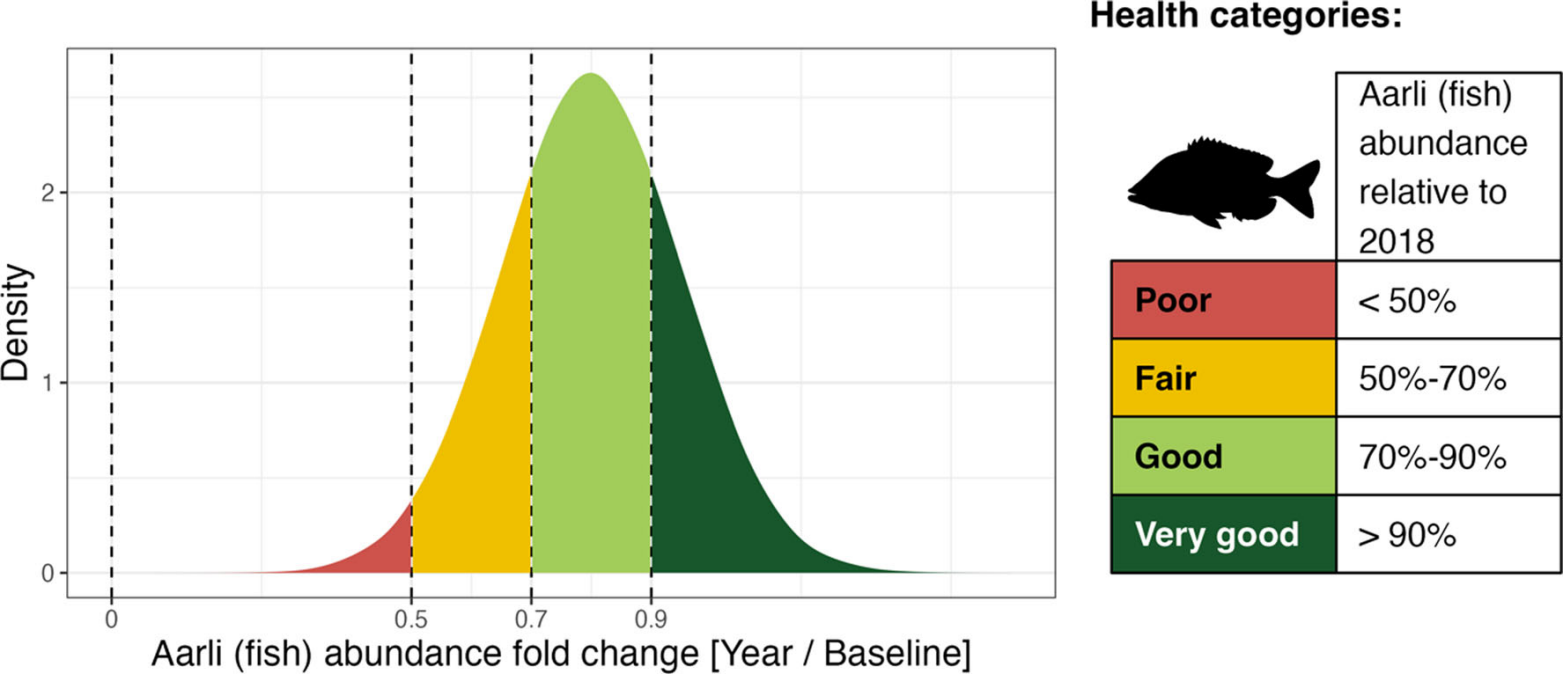
Hypothetical posterior distribution depicting the probability of fish abundance being of a particular health status category (different colours). Fold changes are calculated from the posterior distribution of year-specific estimates of fish abundance, as explained in the main text. The panel on the right shows the health categories assigned by the Traditional Owners (TOs) in the IPA Management Plan

For fish, monitoring indicators are the number of culturally important fish and the diversity of fish species; this case study focuses on the former. Before working in partnership with AIMS, these indicators were evaluated based on a qualitative estimate and compared to a 2011 baseline when the IPA Management Plan was created; for example, the *poor* category meant a limited amount of fish were available. Currently, indicators are evaluated using video stations as a method for quantifying fish abundance and diversity, and results are compared annually to a 2018 baseline corresponding to the first year for which data are available. The change from qualitative to quantitative metrics for health categories, was accompanied by a new system for the ranges used to place *aarli* (fish) into health categories (see Fig. 3). Ranges were selected to combine advice from scientists, pre-existing qualitative categories, and the opinions of rangers and IPA managers. We acknowledge that these would ideally be selected from a priori data that encompasses natural variation in fish populations at this location and may require revision in the future.

As part of designing a monitoring plan, the Bardi Jawi Rangers and AIMS have made every effort to include sites along the extent of Bardi Jawi sea Country, so that the various clan groups can be informed as to what is occurring in their respective areas. However, the IPA Management Plan is an overall plan for all sites collectively, meaning that Bardi Jawi sea Country, and not individual sites, is the reporting level.

### Statistical analyses

#### Main model

This analysis models the sum abundance (*MaxN*)—hereafter simply $A$, collected via western science methods, of the ten fish species selected as important for traditional and recreational fisheries in Bardi Jawi sea Country. This corresponds to the indicator ‘number of culturally important fish’ being monitored as part of IPA management. We model $A$ following key assumptions that were primarily guided by the Bardi Jawi management plan, which envisions monitoring key indicators of fish populations for the entire Bardi Jawi sea Country, rather than specific sites. Towards that goal, we assumed that: (1) sites are a random representation of an overall Bardi Jawi mean fish abundance; (2) the first year of monitoring (2018) is considered the baseline year from which the status of fish abundance health is calculated for subsequent years (2019–2022); (3) the period between 2018 and 2022 represents background natural variation in the absence of any known disturbance, and therefore years are a random representation which should depict natural background variation in fish abundance; (4) video stations are considered to be spatially fixed among years.

Fish abundance (integer counts) data, $A$, often exhibit high spatiotemporal variation (Sale 1978; Sale and Douglas 1984; Holbrook et al. 1994; Anderson and Millar 2004; Cure et al. 2018) which can lead to over-dispersion in the data (Fig. S1). Therefore, we assume that the data are generated by a Negative Binomial process, $NB$:

1 $$\begin{gathered} A \sim NB\left( {\mu , \varphi } \right) \hfill \\ \ln \left( \mu \right) = \beta_{0} + \Delta_{B} + \Delta_{S} + \Delta_{Y} + \Delta_{S:Y} \hfill \\ \beta_{0} \sim {\mathcal{N}}\left( {0, 1} \right); \hfill \\ \Delta_{B} = \zeta_{B} \sigma_{{\Delta_{B} }} ; \zeta_{B} \sim {\mathcal{N}}\left( {0, 1} \right); \sigma_{{\Delta_{B} }} \sim \Gamma \left( {2, 2} \right); \hfill \\ \Delta_{S} = \zeta_{S} \sigma_{{\Delta_{S} }} ; \zeta_{S} \sim {\mathcal{N}}\left( {0, 1} \right); \sigma_{{\Delta_{S} }} \sim \Gamma \left( {2, 2} \right); \hfill \\ \Delta_{Y} = \zeta_{Y} \sigma_{{\Delta_{Y} }} ; \zeta_{Y} \sim {\mathcal{N}}\left( {0, 1} \right); \sigma_{{\Delta_{Y} }} \sim \Gamma \left( {2, 2} \right); \hfill \\ \Delta_{S:Y} = \zeta_{S:Y} \sigma_{{\Delta_{S:Y} }} ; \zeta_{S:Y} \sim {\mathcal{N}}\left( {0, 1} \right); \sigma_{{\Delta_{S:Y} }} \sim \Gamma \left( {2, 2} \right); \hfill \\ \varphi \sim \Gamma \left( {2, 1} \right), \hfill \\ \end{gathered}$$

where ${\beta }_{0}$ is the “global” Bardi Jawi among-sites and among-years mean fish abundance on the natural log scale, ${\Delta }_{[B,S,Y,S:Y]}$ are respectively video stations-, sites-, year- and site-year-specific deviations from ${\beta }_{0}$, and $\varphi$ is the over-dispersion parameter. All ${\Delta }_{*}$ parameters were estimated indirectly as the multiplication of the standardised effects ${\zeta }_{*}$ and their respective standard deviations ${\sigma }_{*}$. Priors were weakly informative relative to the model linear scale (natural logarithm; Fig. S2 in Appendix S2). The prior sampling distributions are the Gaussian ($\mathcal{N}$(mean, standard deviation)) and Gamma ($\Gamma$(shape, inverse scale)). We include the site-year hierarchical interaction term, ${\Delta }_{S:Y}$, to account for any site-specific temporal idiosyncrasies, and because this allows us to calculate site- and year-specific fish abundance means in addition to the overall mean ${\beta }_{0}$ originally included as a management indicator. Importantly, these hierarchical effects also allow us to directly derive indicators from year-specific posterior distributions. For example, ${e}^{{\beta }_{0}+{\Delta }_{Y=2019}}$/${e}^{{\beta }_{0}+{\Delta }_{Y=2018}}$ provides a full posterior distribution of 2019-to-baseline fish abundance ratios that can be directly mapped to the health status categories of the IPA Management Plan (Fig. 3). Moreover, different areas of the posterior distribution, each corresponding to a different category, can be integrated to yield a status credibility (Fig. 3). For example, in Fig. 3 the status of the fish abundance is most likely *good*, although there is some possibility it is either *fair* or *very good*. Status of *poor* has a low credibility. Finally, additional covariates (current, visibility, time of day, tidal height, and depth) were considered in the model as fixed effects, but they did not improve the model fit and were therefore discarded (see Appendix S1 for model selection procedures).

The dataset contained 108 observations of fish abundance on coral reef habitat, $A$ (two video stations had an obstructed field of view and were deemed unsuitable for analyses). The model was fitted under a Bayesian framework using the package brms version 2.20.3 (Bürkner 2017) in R version 4.3.0 (R Core Team 2021) to determine posterior distributions and associated 95% highest posterior density intervals (HDI) for the fitted parameters. The posterior distributions of model parameters (Table 2) were estimated from a total of 10,000 draws—model fitting specifications are provided in Appendix S2. We also checked chain convergence (Fig. S3), ran posterior predictive checks (Fig. S4), and assessed model goodness-of-fit via the Bayesian *R*^2^ (Gelman et al. 2019).

**Table 2:** Model estimates using Bayesian methods. Parameter names correspond to those in Eq. 1

Parameter	Mean estimate	S.D	L-95% HDI	U-95% HDI	$\widehat{R}$
${e}^{{\beta }_{0}}$	13.50	5.33	2.98	23.59	1
${\sigma }_{{\Delta }_{S}}$	0.62	0.40	0.03	1.41	1
${\sigma }_{{\Delta }_{Y}}$	0.38	0.29	0.01	0.94	1
${\sigma }_{{\Delta }_{S:Y}}$	0.48	0.15	0.19	0.77	1
${\sigma }_{{\Delta }_{B}}$	0.22	0.11	0.02	0.43	1
$\varphi$	1.82	0.30	1.26	2.43	1

Lower and upper 95% highest density intervals as well as standard deviation were calculated from posterior distributions. The Gelman-Rubin statistic (Gelman and Rubin 1992), $\widehat{R}$, shows that all four chains have converged

#### Evaluation of monitoring design

To evaluate the effectiveness of the current design in terms of detecting changes in fish abundance over time, we ran a power simulation considering increasing sampling effort ($\alpha$ = {5, 10, 20} samples, but keeping the number of sites fixed) and different levels of multiplicative declines relative to baseline ($\rho$ = {0.05, 0.25, 0.5, 0.7, 0.9, 1}) in a new year. We employed 500 draws from the existing posterior distributions of model parameters in Eq. (1) to simulate datasets with five years of non-disturbance in fish abundance (similar to original data), and a new year where a decline effect is applied to the mean baseline Bardi Jawi fish abundance, e.g., ${e}^{{\beta }_{0}+{\Delta }_{Y=2018}}\times \rho$. The approach can be formalised in four steps:

Step 1: Simulate non-disturbed data between 2018 and 2022

$$A^{\prime}_{1} ,A^{\prime}_{2} ,...,A^{\prime}_{64} \sim NB\left( {\mu , \varphi } \right).$$

Step 2: Simulate disturbed data for a new year with a ${j}^{th}$ decline relative to baseline, $\rho$, and a ${k}^{th}$ sampling effort, $\alpha$.

$${\Delta }^{*}_{Y} \sim {\mathcal{N}}\left( {0, \sigma_{{{\Delta }_{Y} }} } \right)$$

$${\Delta }^{*}_{S:Y} \sim {\mathcal{N}}\left( { 0, \sigma_{{{\Delta }_{S:Y} }} } \right)$$

$${\Delta }^{*}_{{B,\alpha_{k} }} \sim {\mathcal{N}}\left( {0, \sigma_{{{\Delta }_{B} }} } \right)$$

$$\ln (\mu_{j,k}^{*} ) = \ln \left( {e^{{\beta_{0} + \Delta_{Y = 2018} }} \rho_{j} } \right) + \Delta_{S} + \Delta^{*}_{Y} + \Delta^{*}_{S:Y} + \Delta^{*}_{{B,\alpha_{k} }}$$

$$A^{*} \sim NB\left( {\mu_{j,k}^{*} , \varphi } \right).$$

It is important to note that sample-attributable deviations, ${{\Delta }^{*}}_{B,{\alpha }_{k}}$, were only simulated for the additional video samples when $\alpha$ = {10, 20}, i.e., we only simulated 5 and 15 values of ${{\Delta }^{*}}_{B,{\alpha }_{k}}$, respectively. The original 5 ${\Delta }_{B}$ estimated in Eq. (1) were used in all scenarios assuming the location of the original 5 video stations per site remained constant.

Step 3: Concatenate simulated responses, ${A}^{{\prime}{\prime}}=\{{A}^{\prime}, {A}^{*}\}$.

Step 4: Evaluate a modified version of model in Eq. 1 which has an added dummy vector, $X$, to the linear predictor representing “before” (0, 2018–2022) and “after” (1, new year) states,

$$A^{\prime\prime}\sim NB\left( {\nu , \varphi } \right)$$

$${\text{ln}}\left( \nu \right) = {\text{ln}}\left( \mu \right) + \beta_{1} X.$$

This four-step approach allows to test for the probability of ${\beta }_{1}$ being negative, i.e., ${\text{E}}[{\text{I}}\left(({\beta }_{1}|D)<0\right)]$ (i.e., the hypothesis, where $D$ is the data). Steps 1–4 were repeated 500 times for each combination of sampling effort (3 levels) and declines (6 levels), each for a different posterior draw from parameters in Eq. (1), totalling 500 $\times$ 18 = 9,000 simulated datasets and model runs. For each scenario, we then computed the average probability of ${\beta }_{1}$ being negative across 500 simulations as a measure of statistical power. Moreover, at each iteration we evaluated the expectation $\rho = {e}^{{\beta }_{0}+{\Delta }_{Y=new year}}$/${e}^{{\beta }_{0}+{\Delta }_{Y=2018}}$, i.e., the expectation of getting the simulated health category from the estimated decline, by integrating the different regions of its posterior distribution in relation to the IPA Management Plan. Model fitting specifications follow the same as those described above for Eq. (1).

#### Sharing results on-Country

As part of the annual monitoring work with the Bardi Jawi Rangers, a time was set aside on-Country to share and discuss results—*yarning*. This time is crucial for allowing cross-cultural ecological understanding, developing relationships, and testing different ways to communicate scientific results (Davies et al. 2020; Fischer et al. 2022). Discussions were centred around informative presentation formats, simplified explanations of underlying data analyses, and what we could understand from current results regarding changes in fish community metrics. Complementary to this, communications were also extended to the broader Bardi Jawi community, including Elders and decision-makers, children via a one-week workshop with the local school, presentations to the Bardi Jawi Steering Committee, production of short films, and co-presentations between rangers and scientists at national science conferences. During these activities spanning the five years of the program, scientists trialled a variety of communication methods and styles in digital and print media, such as ArcGIS Story Maps, Powerpoint presentations, posters, report cards, and mural artwork. Scientists kept detailed notes on the level of engagement and feedback received, to qualitatively evaluate how the results from this monitoring program have been understood, interpreted, and accepted by the Bardi Jawi Rangers and other members of the community (see Appendix S3 for a summary). Feedback was used to develop the format and informational content of science communication products for the monitoring program. We acknowledge that these views are centred on the perceptions of western scientists; a more systematic way of recording evidence directly from the Traditional Owners to formally test their understanding of the information being communicated, will be the next step to improve the conclusions from this work.

## Results

### Main model

**Fig. 4:**
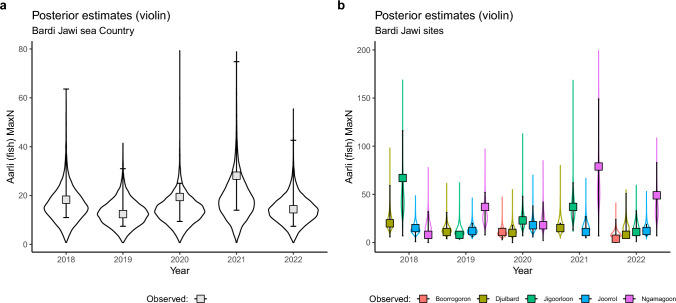
*MaxN* combined across the 10 important fish species/groups in Bardi Jawi Country. **a** shows the median trends per year for Bardi Jawi sea Country, whereas **b** shows trends per site. Symbols (median) and error bars (95% HDI) were calculated from data, whereas violin plots depict posterior distributions estimated by the model described in Eq. (1)

Our statistical model explained 36% of the variation observed in the data (Bayesian R^2^ 95% highest posterior density interval (HDI): 17%–56%). The mean combined *MaxN* of the 10 important fish species/groups—${e}^{{\beta }_{0}}$—in Bardi Jawi Country was 13.5 fish across all sites and years, although this estimate was quite uncertain (HDI: 3.0–23.6; Fig. 4; Table 2). This uncertainty was also reflected in how much *MaxN* varied across space and time: 1.6-fold (i.e., ${e}^{2\times {\sigma }_{{\Delta }_{B}}};$ HDI: 1.0–2.4) across video samples, 6.4-fold (i.e., ${e}^{2\times {\sigma }_{{\Delta }_{S}}};$ HDI: 1.0–16.2) across sites, 2.9-fold (i.e., ${e}^{2\times {\sigma }_{{\Delta }_{Y}}};$ HDI: 1.0–6.5) across years, and 2.7-fold (i.e., ${e}^{2\times {\sigma }_{{\Delta }_{S:Y}}};$ HDI: 1.3–4.4) across sites and years. The data exhibited a moderate degree of over-dispersion (mean $\varphi$ = 1.8; HDI: 1.3–2.4).

**Fig. 5:**
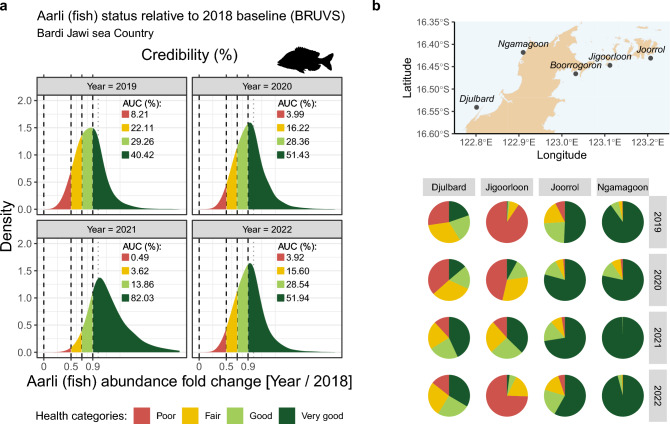
Overall (**a**), and site-specific (**b**) health status of *MaxN* combined across the 10 most important fish species/groups in Bardi Jawi sea Country. Health status classifications follow the health categories in the Bardi Jawi IPA Management Plan. Density plots in (**a**) depict the credibility of each category based on posterior distributions of abundance fold change (${e}^{{\beta }_{0}+{\Delta }_{Y=\{2019:2022\}}}$/${e}^{{\beta }_{0}+{\Delta }_{Y=2018}}$), with credibility being calculated as the relative area under the curve (AUC; in %). The vertical grey dotted line at 1 indicates no change. In (**b**), credibility estimates were re-expressed as pie charts for site-specific calculations (monitored sites indicated on the map), i.e., incorporating ${\Delta }_{S}+{\Delta }_{S:Y}$ into the calculations. The site Boorrogoron is not displayed because it was only sampled in 2020 and 2022, and therefore we could not back-calculate changes relative to the baseline year, 2018

Overall, the sum of *MaxN* from the 10 important fish species/groups oscillated around the baseline median for Bardi Jawi sea Country combined (Fig. 4a). Based on the posterior distributions of fold change relative to the baseline year 2018 for Bardi Jawi sea Country, there was a 75%, 65%, 32% and 65% chance the fish abundance had declined (the proportion of the posterior below 1) in 2019, 2020, 2021 and 2022, respectively (Fig. 5a). However, based on the proportion of the posterior showing either an increase, or small decline (< 10%) in the population, this indicator was selected as *very good* throughout 2019–2022, although with a 40.4–82% credibility (see Fig. 5a).

An overall decline was observed for most sites, except for Ngamagoon which has been increasing (Fig. 4b). These trends were translated into varied health statuses across sites and years (Fig. 5b). For example, whereas sites Djulbard and Jigoorloon exhibited mostly a *poor* or *fair* status in most years, Ngamagoon and Joorrol exhibited high credibility for a *very good* status (Fig. 5b).

### Evaluation of monitoring design

**Fig. 6:**
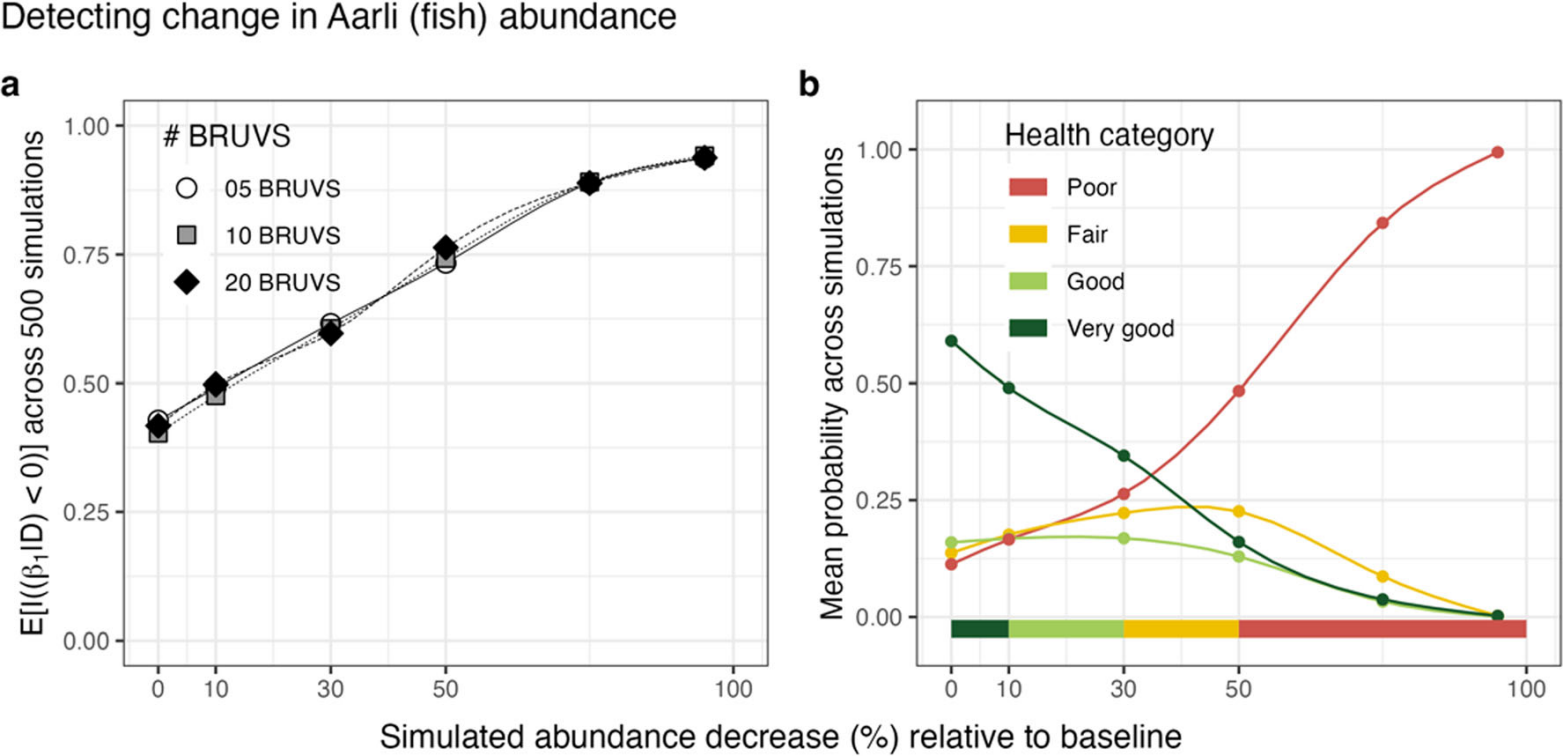
Results of simulations used to evaluate monitoring design. **a** shows the average probability (combined across 500 Bayesian simulations) to detect a negative decline in fish abundance at a new year (see Evaluation of monitoring design in the Methods section for a full description of the approach) for three different sampling efforts (i.e., number of samples). **b** shows the mean probabilities of each health indicator category (coloured points and lines) across the simulated population decline gradient. The x axis depicts the different values of $\rho$—re-expressed as a percentage decline—used for the simulations. Different sections of the x axis (horizontal colour bar at the bottom) encompass the IPA Management Plan indicator categories. The mean probabilities of each category were calculated from posterior distributions of ${e}^{{\beta }_{0}+{\Delta }_{Y=new year}}$/${e}^{{\beta }_{0}+{\Delta }_{Y=2018}}$. Given the lack of obvious difference among sampling effort scenarios (**a**), (**b**) was drawn from scenarios with only 20 video stations. For visual purposes, connecting lines in **a** and **b** have been smoothed using splines in all panels

Our simulation approach revealed limited capacity of the current monitoring design to detect immediate change over the following monitoring year, regardless of the sampling effort adopted (Fig. 6a). Specifically, an > 80% credibility to detect change was only observed once 50–70% of the fish population were removed from 2018 baseline model estimates, and more moderate declines such as 30% yielded a credibility of ~ 60%; thus, the overall credibility was slightly larger for decline than increase in fish population abundance. Moreover, the simulation that imposed no change to fish abundance relative to baseline (0% decline in Fig. 6a) yielded ~ 40% credibility of a negative decline across the different efforts. However, the expectation for a simulation that recovers a true (simulated) mean of 0% change, on average, should be 50%, i.e., half of the combined posterior distributions should be negative and the other half positive.

The uncertainty in the posterior distributions combined across the different simulations resulted in large uncertainty for the different health categories (Fig. 6b). For example, contrary to expectation (bottom colour bar in Fig. 6b), simulated declines of 10–50% did not translate into high credibility of *fair* and *good* status, however their credibility should have been higher if the modelled simulated data (Step 4 in the Methods section for Evaluation of monitoring design) yielded parameter values close to the original simulation process (Steps 1–3 in the Methods section for Evaluation of monitoring design).

## Discussion

Here, we explored data collected by the co-designed environmental monitoring partnership between the Bardi Jawi people and AIMS. We specifically explored a Bayesian framework approach to estimate uncertainty and more fully communicate monitoring trends from western science to Indigenous people by: (1) providing an indication of the degree of uncertainty in the change estimates calculated in the results, and (2) facilitating a better understanding of the limitations of monitoring for identifying sudden small changes in fish community metrics. We contend that presenting estimated changes in monitored environmental parameters (e.g., fish population abundance) together with a numeric probability statement that aligns with a simple traffic-light system, can improve decision-making processes for sea Country managers (Budescu et al. 1988). We first discuss our overall results and modelling limitations, and then turn to potential general implications of our work to Indigenous monitoring and better ways that western science can simultaneously learn from and contribute to it.

### Main model

Our model captured 38% of the variation in the data mostly via its hierarchical structure. Characterising and determining fish abundance dynamics has been a topic of much debate for decades (Sale 1978; Sale and Douglas 1984; Anderson and Millar 2004; Irigoyen et al. 2013; Thibaut and Connolly 2020). It would be currently speculative to try and connect the changes in abundance observed here to a specific environmental cause or to differences in fishing intensity potentially associated with improved access to Bardi Jawi sea Country following road sealing in 2019. Our temporal dataset is limited in duration, there are currently no records of fishing or visitation pressure in the area, and we need to further explore potential variables that can help explain abundance variations.

Adding covariates such as time of the day, tidal height and depth did not improve the model predictive capacity (Appendix S1). However, we anticipate further improvements to covariates measured and our data modelling capacity to help explain the factors behind fish abundance variation, increase the data variability explained and provide more accurate results with greater credibility and less uncertainty. For example, one area of improvement is the addition of environmental covariates that could help explain more of the data in a deterministic way; for example, temperature, chlorophyl-a (proxy for productivity) and habitat complexity. It is also unclear whether the Negative Binomial distribution—although the most suitable based on the nature of the data—is the true underlying distribution that governs fish abundance in space and time.

### Evaluation of monitoring design

The probability of the current monitoring design detecting change was better than chance odds (50/50) only once > 10% of the fish population was removed, and our inability to detect a useful effect size with reasonable certainty raises at least four questions for this monitoring program going forwards.

First, is power to detect change limited by the length of our time series? Five years of data may not sufficiently encompass the spatiotemporal variation of fish communities in the highly variable coastal environment of the Kimberley. Longer datasets may be required to properly account for and override natural variation in space and time so that trends in abundance and the effects of disturbance, are more accurately identified (Magurran et al. 2010; Lindenmayer and Cunningham 2011). Identifying the minimum number of monitoring years for this to occur is essential information for the design of any monitoring program and setting expectations of what western science approaches can and cannot do in the service of marine resource management.

Second, could the power to detect change with greater certainty be improved by increasing the sampling effort? Our results indicate that increasing the number of video stations by up to four times at a particular site, will have no impact on improving the power to detect change. This means that a larger number of samples would not reduce variability in our estimates of fish abundance. There are two potential reasons for this. Firstly, our indicator metric (*MaxN* for ten culturally important fishes) includes species with different lifestyles (site-attached benthic species, roving schools, and pelagics) and different levels of attraction to bait (herbivores and carnivores), so an assessment may need to be made separately for each of these groups. Secondly, high environmental variation results in highly variable fish communities at very small spatial scales (250–500 m). Care was taken to minimize variation by being as consistent as possible in sample location, habitat, time of year, time of day, tide, and visibility. However, these factors may vary drastically in the Kimberley even within the bounds of our efforts for consistency and could be preventing us from detecting smaller declines in abundance.

Third, should we simulate changes for specific sites? Although “the capacity (or power) to detect change” may seem like a relatively straightforward, innocuous question, we note that power is tied to a precise hypothesis (i.e., model structure) and therefore a simulation algorithm (in the case of multilevel models; Kain et al. 2015). For example, simulating change in only one site while treating the remaining sites as controls, or adding more post-disturbance years, might have improved our power substantially (e.g., simulating a spatially expanded BACI design; Underwood 1994). However, we note that the power simulation exercised here was constructed to service the IPA plan which is focused on Bardi Jawi as a single reporting unit, and would be primarily concerned with understanding the power to detect change following an immediate disturbance pulse.

Lastly, where do we go from here? The aim of the Bardi Jawi people is to reduce threats to culturally important fish species and improve their abundance to guarantee food security for their community. Therefore, for this monitoring program to be fit-for-purpose, we need to further explore options to improve the power to detect change in fish abundance through time with credibility. This may involve modelling fish abundance with metrics other than *MaxN* (Schobernd et al. 2014; Sherman et al. 2018), collecting more fine-scale environmental data using loggers attached to video stations, re-thinking how change is evaluated (i.e., comparison to baseline vs. moving mean, and specific ranges of health categories), or expanding the spatial extent of the current sampling design. The latter is currently being considered with the recent creation of the Bardi Jawi Gaarra Marine Park co-managed by Bardi Jawi and the Western Australian state government. An adaptive monitoring approach informed by the current dataset to amend the current sampling design may be possible without losing the integrity of the current dataset (Lindenmayer et al. 2011). Workshopping this approach together with researchers, TOs, and state government park managers will be key to its success.

### Decision making with uncertainty

Communication of monitoring results to non-scientific audiences involves sharing a simple message on the health status of a resource. In most cases, this involves presenting temporal change as a trend—increase, decrease, no change or, in this example, as a health category. This simple message is then used by decision-makers and managers to implement strategic changes or maintain the status quo. In most cases, this simplification of message requires scientists to exclude associated uncertainty. However, understanding this uncertainty is key to decision-making (Pople et al. 2007).

**Fig. 7:**
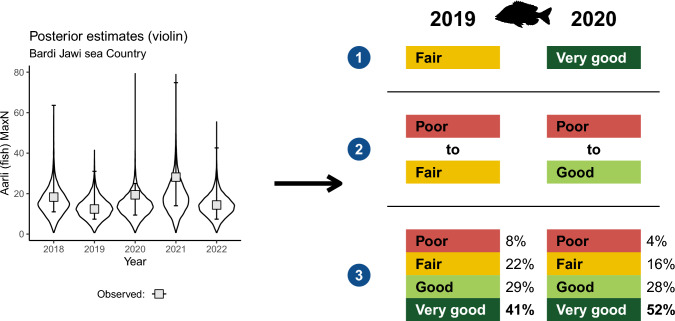
Representation of three possible communication options from monitoring data collected in the Bardi Jawi Rangers-AIMS monitoring partnership. The plot on the left is redrawn from Fig. 4a and used as an example. On the right, (1) takes only the median value into account, and yields 68% and 106% of the abundance relative to the 2018 baseline; therefore, *fair* and *very good* would be the health categories reported for 2019 and 2020; (2) introduces uncertainty associated with the median estimate (based on the observed HDIs), so that a series of health categories could be possible, and (3) presents Bayesian estimates of credibility (% of AUC) for each possible category in the IPA Management Plan (see Fig. 5a). We contend that option 3 is more informative, provides added credibility and is better suited to decision-making

We have found that providing Bayesian probability estimates for all health categories in the Bardi Jawi IPA Management Plan, offers additional information to communicate both uncertainty and credibility in estimates, providing a stronger base for more accurate decision-making from monitoring data (see Fig. 7). Using only median values without incorporating uncertainty (Fig. 7, scenario 1), results in selection of a unique health category for an indicator (i.e., *fair*). While a simple approach to communicate, the more accurate representation of reality is more complex due to uncertainty. Incorporating highest density intervals calculated from the raw data reveals that the true health category can lie between at least a couple of selections (Fig. 7, scenario 2). Finally, by incorporating probabilities from Bayesian posterior distributions, we can provide a fully transparent appraisal, conditioned on the data and the model, of each health category being selected for a particular indicator (Fig. 7, scenario 3). With this information, sea Country managers still would select the single health category with the highest percentage—in this case *very good*, while getting information on the relative risk of that selection across all health categories. In this example, it is most likely that either *good* or *very good* is selected for the ten most important fish species, with *poor* selection being unlikely (< 10%). In a scenario where probabilities are equal across categories, managers may need to be on higher alert. Importantly, by including spatial hierarchies in our models, we can further explore these probabilities at the site level and focus conservation effort where it is most needed. In this example, the indicated decline at sites Djulbard and Jigoorloon should be closely watched (see Fig. 5b).

Better understanding of the uncertainty associated with our long-term estimates of fish abundance has also led to re-thinking the appropriateness of the quantitative ranges selected for existing health categories to evaluate resource status. For example, although a goal for the Bardi Jawi people is to have abundant fish species, the best *very good* category still allows for a 10% decline in abundance. Furthermore, simulations of population decline gradients are not being reliably identified and placed into the appropriate health category. It may be more appropriate to adjust the ranges used to place *aarli* (fish) in the health categories; for example, to include increasing trends (> 100% of baseline), and more reliably identify population decline gradients triggering a management response. These are evolving questions that will require refining over time in collaboration with rangers and TOs, with current results guiding us into the future.

### Moving forward

Despite the uncertainties in rapidly and sensitively detecting changes in the local fish community, the Bardi Jawi community have embraced the monitoring program and commitment to the collaboration remains strong. The results presented here are part of an important journey of understanding for both parties. For the western science practitioners, it provides an opportunity to understand where sampling improvements can be made and how to best communicate monitoring results to a non-scientific community. For TOs, it highlights that western science has its own limitations, and that science is a journey of discovery rather than a definitive destination. For both, it represents an opportunity to come together, combine Traditional Ecological Knowledge with western science, and collaborate to improve the program. There is a strong understanding from both knowledge bases that ecosystems are inherently under constant change and that the reasons for this are often complicated and not easily understood. On-going visits to Country to re-engage across all members of community is integral to this broadening of sea Country knowledge and its future management.

Explaining the complex mathematics behind Bayesian modelling is a more difficult concept. Rangers and community are most comfortable with results presented as annual means connected via trendlines. When talking to these graphs during meetings and presentations, they focus on the mean value and the trend it shows with respect to previous years but are now embracing uncertainty in these estimates as part of the discussion. Presentation of Bayesian credibility statistics (% area under the curve) as pie charts divided into the different colours of the traffic light system has been included in monitoring results communications with the Bardi Jawi Rangers to add further insights into how and why health categories are selected. Although there is a strong need for the selection of a single rating from this traffic light system showing resource status that can be easily shared with community, these pie charts can better inform rangers and managers on the true nature of ecosystem resources. Further on-Country *yarning* to link this uncertainty to the decision-making process is planned for future work.

For the time being, the monitoring program continues to supply information on the status of important food sources for the community. This information is being used to guide management decisions for the IPA, inform traditional fisheries management practices, and influence policy. For example, monitoring result outputs were used by the Bardi Jawi Rangers and other TOs in recent negotiations for co-designing and zoning the new Bardi Jawi Gaarra Marine Park in their sea Country. No decision-making process regarding resource status or harvest quotas for traditional fisheries has yet occurred because of this work. The Bardi Jawi people have a long history of managing their fisheries using traditional methods which mostly involve seasonal catch restrictions, with catch being limited to when fish are fat (Rouja et al. 2003). Moreover, results from this monitoring program together with observations on Country, indicate that fish populations are generally healthy. However, with increasing pressures to Bardi Jawi sea Country (e.g., increased recreational fishing, climate change), monitoring data may play an increasingly important role in the future to guide new management policies for both the IPA and the marine park.

Long-term ecological monitoring is key to evidence-based environmental policy, decision making and management. Keeping track of resources over long timeframes is already understood and deeply embedded in Aboriginal and Torres Strait Islander’s Traditional Ecological Knowledge. It has formed the basis for an intricate knowledge system to understand natural patterns based on long-term observations of when resources can be harvested. With current changes in increasing population, climate change, species extinction and restoration activities amongst others, long-term evidence-based monitoring is key to evaluating effects of change and developing ecologically sustainable resource management strategies that promote ecological and social wellbeing. We envision that the lessons learnt from this work will guide TOs, scientists, and managers into better-designed and fit-for-purpose monitoring work that can help support these goals. Therefore, we conclude by providing the following guidelines to assist in developing a robust joint monitoring partnership that values all ecological knowledge: (1) manage expectations—neither western science nor Traditional Ecological Knowledge has all the answers, but together, they are complementary: (2) set out clear intentions of what is being sought from a monitoring program and associated data from the beginning of the partnership: (3) evaluate the sampling plan early with available data and revise/adapt if necessary; (4) discuss uncertainty in long-term estimates regularly and from the beginning; (5) include several indicators for the health status of a resource so as not to rely on a single metric such as *MaxN*, ideally including traditional metrics and data on resource use; and (6) be flexible to adaption in monitoring plans, effort required, and designs, to improve their utility to marine management and conservation outcomes.

### Supplementary Information

Below is the link to the electronic supplementary material.

Supplementary file1 (PDF 2557 kb)
