# Supplementary material for: Incorporating uncertainty in Indigenous sea Country monitoring with Bayesian statistics: Towards more informed decision-making
Source: Ambio. 2024 Feb 14;53(5):746–63. doi: 10.1007/s13280-024-01980-2 (PMC10992390; doi:10.1007/s13280-024-01980-2)
Supplement: Supplementary file 1 — Supplementary file1 (PDF 2557 kb) [file 13280_2024_1980_MOESM1_ESM.pdf]

Ambio

Supplementary Information

This supplementary information has not been peer reviewed.

Title: **Incorporating uncertainty in Indigenous sea Country monitoring with Bayesian statistics: towards more informed decision-making**

Table S1. Details on sites sampled in the Dampier Peninsula using Baited Remote Underwater Video Stations (BRUVS), to estimate fish abundance.

**Site**	**lat**	**long**	**Description**
Djulbard	-16.5394	122.8138	Rocky reef that extends NW from the mouth of the mangrove creek. Can be affected by westerly winds and currents if tide is not properly selected. Sparse coral cover, mixed with macroalgae. Exposed. Reef partially exposed during spring lows; BRUVS locations selected for permanently subtidal.
Ngamagoon	-16.4296	122.9027	Rocky reef following the contour of the coastline starting ~1km north of the mangrove creek entrance. Sparse coral cover mixed with macroalgae - similar to Djulbard. Exposed and not good visibility if western winds and high currents.
Boorrogoron	-16.4659	123.0323	Coral reef site with moderate coral cover. BRUVS follow the contour of the reef and are deployed at the reef margin. Visibility can be low and best targeted at slack tide; nearby muddy habitat close to pearl farm can increase turbidity.
Jigoorloon	-16.4503	123.1057	Coral reef site with high to moderate coral cover. BRUVS follow the contour of the reef and are deployed at the reef margin. Close to boat launch at Ardyaloon.
Joorrol	-16.4312	123.2060	Coral reef site with high coral cover. BRUVS follow the contour of the reef and are deployed at the reef margin. Close to boat launch at Ardyaloon.

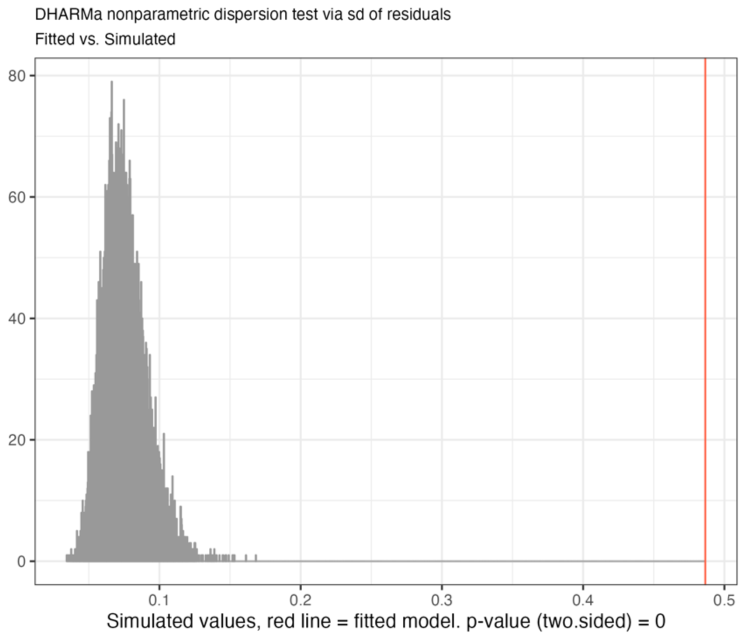

Fig. S1. Non-parametric test for dispersion from posterior simulated residuals of model in equation (1) refitted assuming a Poisson distribution instead of a Negative Binomial. Model validation plot generated using the R package DHARMa version 0.4.6 (Hartig 2022).

Hartig, F (2022). DHARMa: Residual Diagnostics for Hierarchical (Multi-Level / Mixed) Regression Models. R package version 0.4.6. https://CRAN.R-project.org/package=DHARMa

Appendix S1. Selection of model fixed covariates

Covariates were considered for inclusion in modelling to account for variation in fish abundance related to time-of-day (Thompson and Mapstone 2002; Mallet et al. 2016; Zemke-White, J., and K. 2002), depth in the water column (Brokovich et al. 2008; MacDonald, Bridge, and Jones 2016; Dubuc et al. 2019), tidal amplitude (Gibson 2003; Dubuc et al. 2019; Ellis and Bell 2008; Sheaves, Johnston, and Baker 2016), current speed (Fulton, Bellwood, and Wainwright 2005; Liao 2007; Eggertsen, Hammar, and Gullström 2016), and water clarity or visibility (Unksworth et al. 2014). The time and approximate depth of each video station deployment was recorded by rangers during sampling. Time was expressed as the number of minutes past midnight, hereafter M, and the natural log of M was considered for modelling as it conferred greater linarity with the response. The depth in meters, D, was recorded from the boat depth sounder at the time of deployment; it is unlikely to correspond to the exact position of the video station on the ocean floor and is therefore an approximate measure. Measurements of tide, current, and water clarity were approximated using video imagery and tidal predictions. Tidal amplitude, T, was predicted for the time of each deployment using a harmonic tidal clock (XTide 2; Flatter 2022; using harmonics parameters at https://github.com/manimaul/MX-Tides-iOS/blob/master/resources/harmonics-2004-06-14.tcd), with Karrakatta Bay as the reference station (latitude -16.3667, longitude 123.0333; approximately 10 km and 34 km from the nearest and furthest BRUVS deployments). Current speed, C and water clarity, W, were categorized by video analysts using an ordinal scale 0-5 with 0 representing low current speed or water clarity and 5 representing high current speed or water clarity (see Table S2).

Alternative models of A, the $MaxN$ summed over the ten fish species presented in Table 1 of the main text, resulted from the addition of covariates to the intercept only model shown below in equation 1 (eq 1). We considered three alternative models resulting from the inclusion of all covariates (eq 2); $\ln(M)$, D and T (eq 3); and $\ln(M)$ and T (eq 4). Due to overdispersion in A we assumed a Negative Binomial data generating process, NB:

$$A \sim NB(\mu, \varphi)$$

eq 1 $$\ln(\mu) = \beta_0 + \Delta_B + \Delta_S + \Delta_Y + \Delta_{S:Y}$$

eq2 $$\ln(\mu) = \beta_0 + \beta_1 \ln(M) + \beta_2 D + \beta_3 T + \beta_4 C + \beta_5 W + \Delta_B + \Delta_S + \Delta_Y + \Delta_{S:Y}$$

eq3 $$\ln(\mu) = \beta_0 + \beta_1 \ln(M) + \beta_2 D + \beta_3 T + \Delta_B + \Delta_S + \Delta_Y + \Delta_{S:Y}$$

eq4 $$\ln(\mu) = \beta_0 + \beta_1 \ln(M) + \beta_3 T + \Delta_B + \Delta_S + \Delta_Y + \Delta_{S:Y}$$

$$\beta_* \sim \mathcal{N}(0, 1)$$

$$\Delta_* \sim \zeta_* \sigma_{\Delta_*};\ \ \zeta_* \sim \mathcal{N}(0,1);\ \ \sigma_{\Delta_*} \sim \Gamma(2,2);$$

$$\varphi \sim \Gamma(2,1)$$

where β_0 is the "global" Bardi Jawi among-sites and among-years mean fish abundance on the natural log scale; $\beta_{[1,2,3,4,5]}$ are the slope parameters adjusting for $\ln(M)$, D, T, C, and W; $\Delta_{[B,S,Y,S:Y]}$ are the random effects adjusting for BRUVS-, site-, year- and site-year-specific deviations; and φ is the overdispersion parameter. Δ_* parameters were estimated indirectly as the multiplication of the standardised effects ζ_* and their respective standard deviations σ_*. The prior sampling distributions are Gaussian ($\mathcal{N}$(mean, standard deviaton)) and Gamma (Γ(shape, inverse scale)) and were calibrated by prior predictive checks to be weakly informative. See the main text *Statistical Analyses: Main model* for further explanation of modelling and note that the β_1 presented here is unrelated to that used in *Statistical Analyses: Evaluation of monitoring design*.

Alternative models were compared to the intercept-only model using leave-one-out cross-validation (LOO), which is a fully Bayesian model selection procedure for estimating pointwise out-of-sample prediction accuracy (Hooten and Hobbs 2015; Vehtari, Gelman, and Gabry 2016). For each model, we calculated the expected log pointwise predictive density ($\widehat{elpd}_{loo}$) using the log-likelihood evaluated at the posterior simulations of the parameter values (Vehtari, Gelman, and Gabry 2016). The pairwise differences in $\widehat{elpd}_{loo}$ ($\Delta\widehat{elpd}_{loo}$) were compared between models along with their standard errors. The computed $\Delta\widehat{elpd}_{loo}$ values, relative to the intercept only model, are shown in Table S3 where we see that inclusion of covariates to the intercept only model reduced the predictive performance. For this reason, and given its relative simplicity, the intercept-only model was retained.

Table S2. Water clarity and current speed categories (0 to 5) along with water clarity category reference images and current speed category descriptions.

Category	Category reference	
	Current speed	Water clarity
0	No obvious water movement.	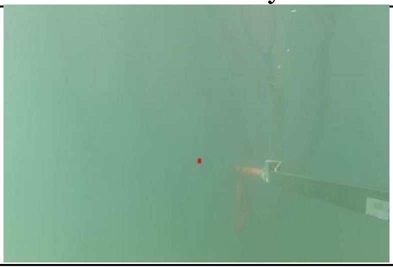

1	Water movement obvious. Material moving very slowly across the screen. Fish not affected. Vegetation may sway slightly.	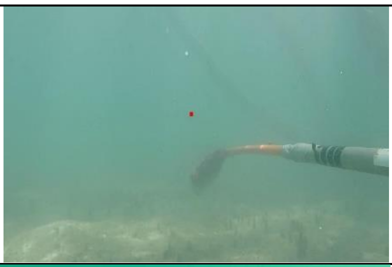
2	Vegetation is clearly swaying, and/or partially leaning in a uniform direction. Particles in the water are moving together horizontally in the water. Fish not affected by current.	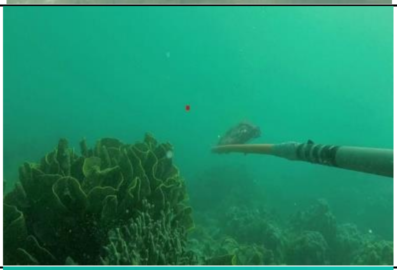
3	Vegetation is swaying strongly and is uniformly/ or partially leaning in one direction with the current. Particles and very small bits of vegetation are in suspension and moving together horizontally in the water. Some fish may be affected by current.	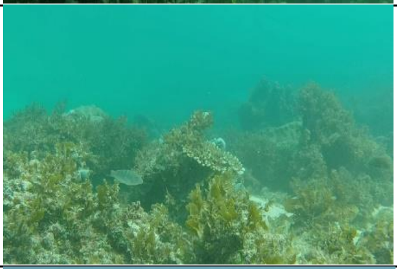
4	All vegetation is leaning all together in one direction at a 45° angle with the current. Particles and small bits of vegetation are moving uniformly horizontally in the water. Fish are affected by current.	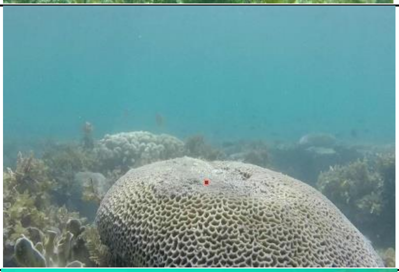
5	Very fast current. All Vegetation is uniformly horizontally flattened to the substrate. Particles and large pieces of vegetation are in suspension and moving very fast in the water. Current has a significant effect on fish.	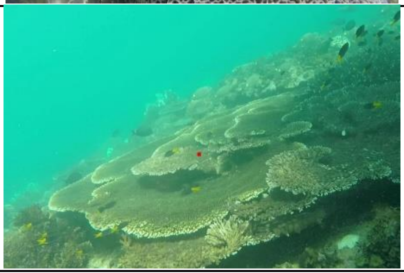

Table S3. Leave-one-out cross-validation (LOO) expected log pointwise predictive density ($\widehat{elpd}_{loo}$); pairwise differences in $\widehat{elpd}_{loo}$ for models with the inclusion of all, or a subset, of the fixed covariates time of day (M), depth in the water column (D), tidal amplitude (T), current speed (C) and water clarity (W), relative to the intercept only model ($\Delta\ \widehat{elpd}_{loo}$); and the respective standard errors.

Model covariates	$\widehat{elpd}_{loo}$	$se(\widehat{elpd}_{loo})$	$\Delta\ \widehat{elpd}_{loo}$	$se(\Delta\ \widehat{elpd}_{loo})$
None (intercept only)	-435.346	10.296	0	0
$\ln(M)$, T	-436.094	10.265	-0.747	0.869
$\ln(M)$, D, T	-437.066	10.659	-1.720	1.738
$\ln(M), D, T, C, W$	-438.682	10.919	-3.335	2

Appendix S2. Model prior and fitting specifications

Priors for the best model were weakly informative relative to the model linear scale (natural logarithm; Fig. S2). We note that some of the standard deviations (σ_*) were influenced by the priors most likely due to current time span of the monitoring program, but we expect this issue to be resolved over time as the program evolves and more data is added.

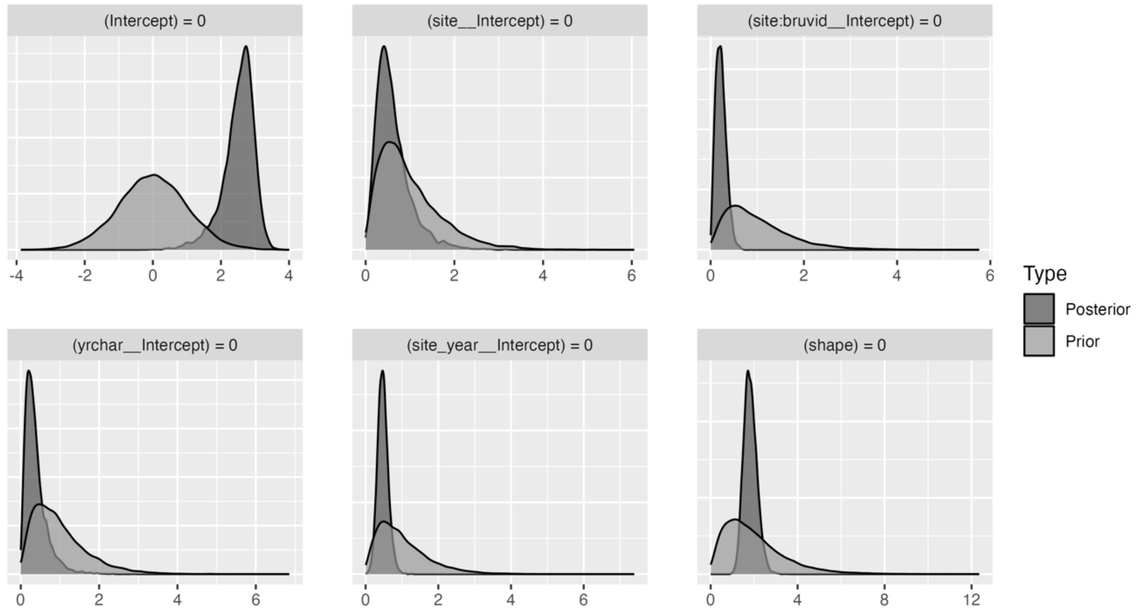

Fig. S2. Visual assessment of prior influence on posterior distribution. Parameter estimates are on the link (natural log) scale.

Parameters were estimated using No-U-Turn Sampler (NUTS) Hamiltonian Monte Carlo (HMC) by constructing four chains of 5,000 steps each. Half of these iterations (2,500) were used as a warm-up, so a total of 10,000 steps were retained to estimate posterior distributions (i.e., 4 × (5,000 - 2,500) = 10,000). All four independent chains reached convergence (Fig. S3), i.e., the Gelman-Rubin statistic (Gelman and Rubin 1992), $\hat{R}$, was approximately 1 for all parameters. We adopted a target average proposal acceptance probability of 0.99, and a maximum tree depth of 20, i.e., the maximum number of steps in each iteration was 220. No divergent transitions were observed. Posterior predictive checks to assess goodness-of-fit are provided below in Fig. S4.

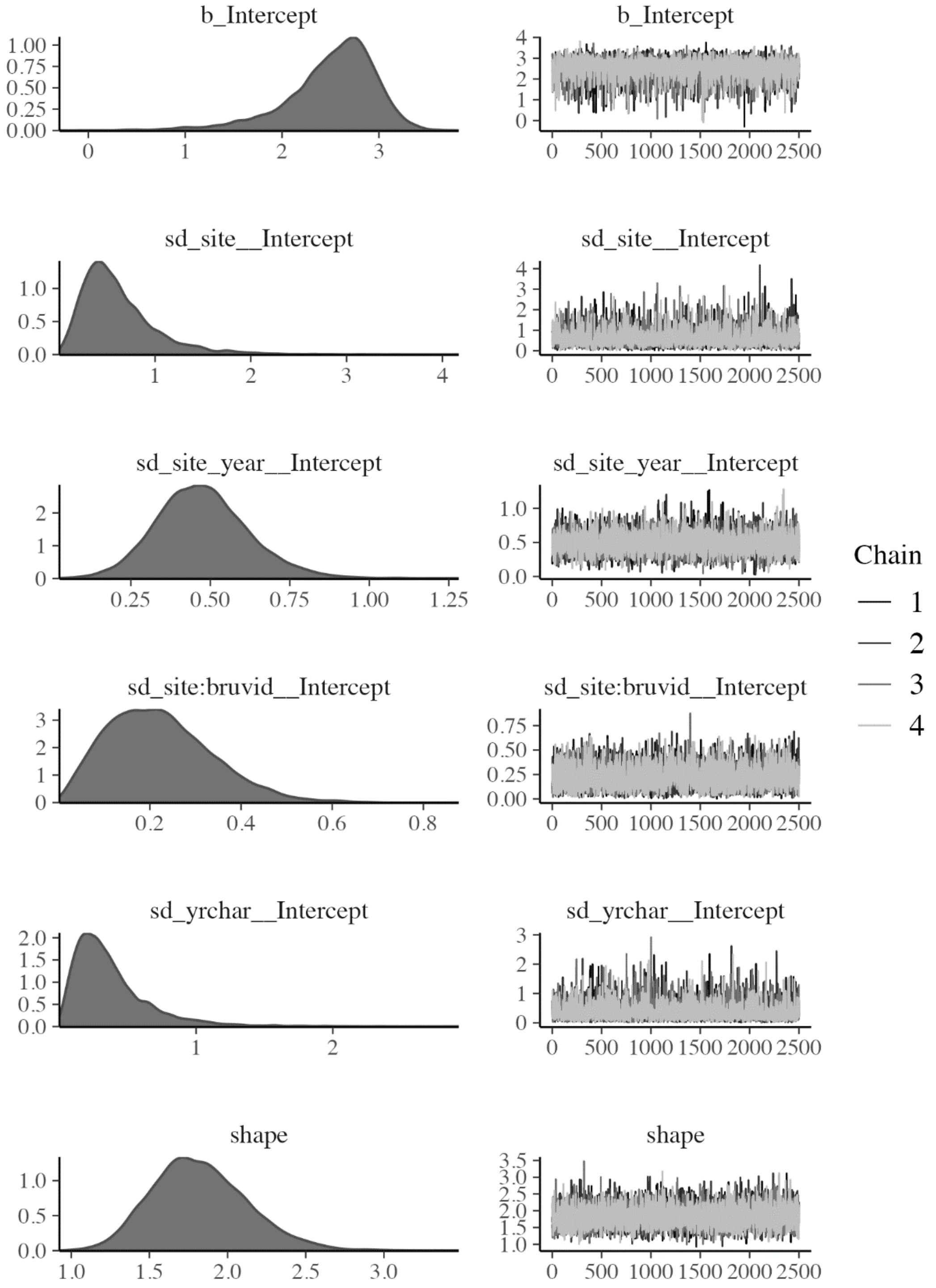

Fig. S3. Left-hand column contains posterior distribution of parameters in the best model (see Appendix S2 below). Parameter estimates are on the link (natural log) scale. Right-hand column contains traceplots demonstrating that all chains converged on the same results.

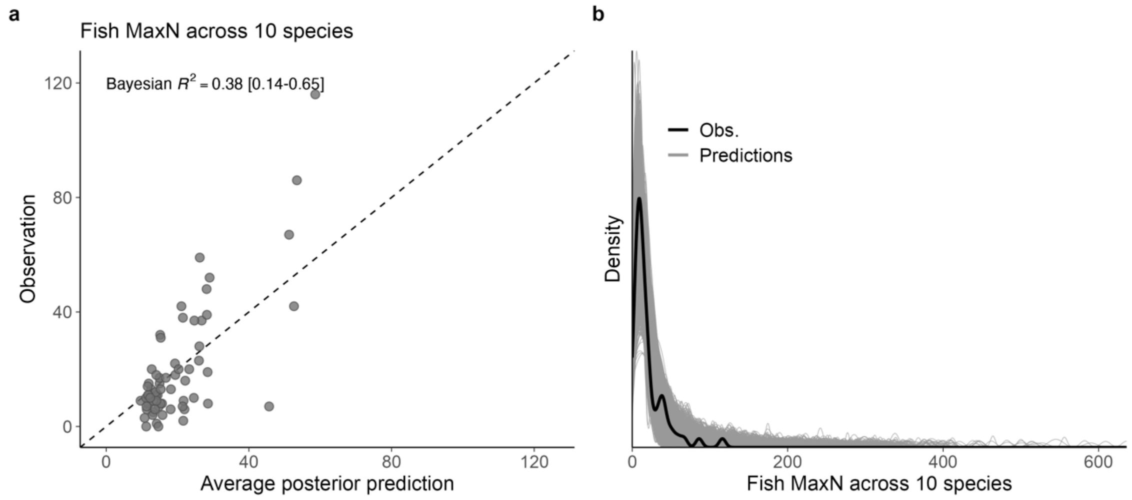

Fig. S4. Posterior predictive checks for main model of equation (1). (a) depicts the relationship between observations and average predictions of the *MaxN* summed across the ten fish species in Table 1. Dashed line represents a 1:1 relationship. In (b), observed density of *MaxN* (thick black line) overlaying 1,000 mean posterior predictions (thin grey lines).

Appendix S3. Summary of "Closing the Circle", an investigation into sharing monitoring results across generations of Traditional Owners in sea Country. Available online at: https://wamsi.org.au/wp-content/uploads/2021/10/Closing-the-Circle-AIMS-Workshop-Report.pdf. This investigation was conducted during a one-week workshop at the One Arm Point Remote Community School with participation of school children, Bardi Jawi and Oorany Rangers, Elders and members from the Bardi Jawi Prescribed Body Corporate and Ranger Steering Committee, as well as researchers from AIMS and The University of Western Australia. This work was done in collaboration with the Western Australian Marine Science Institution, Scitech, and Inspiring Australia.

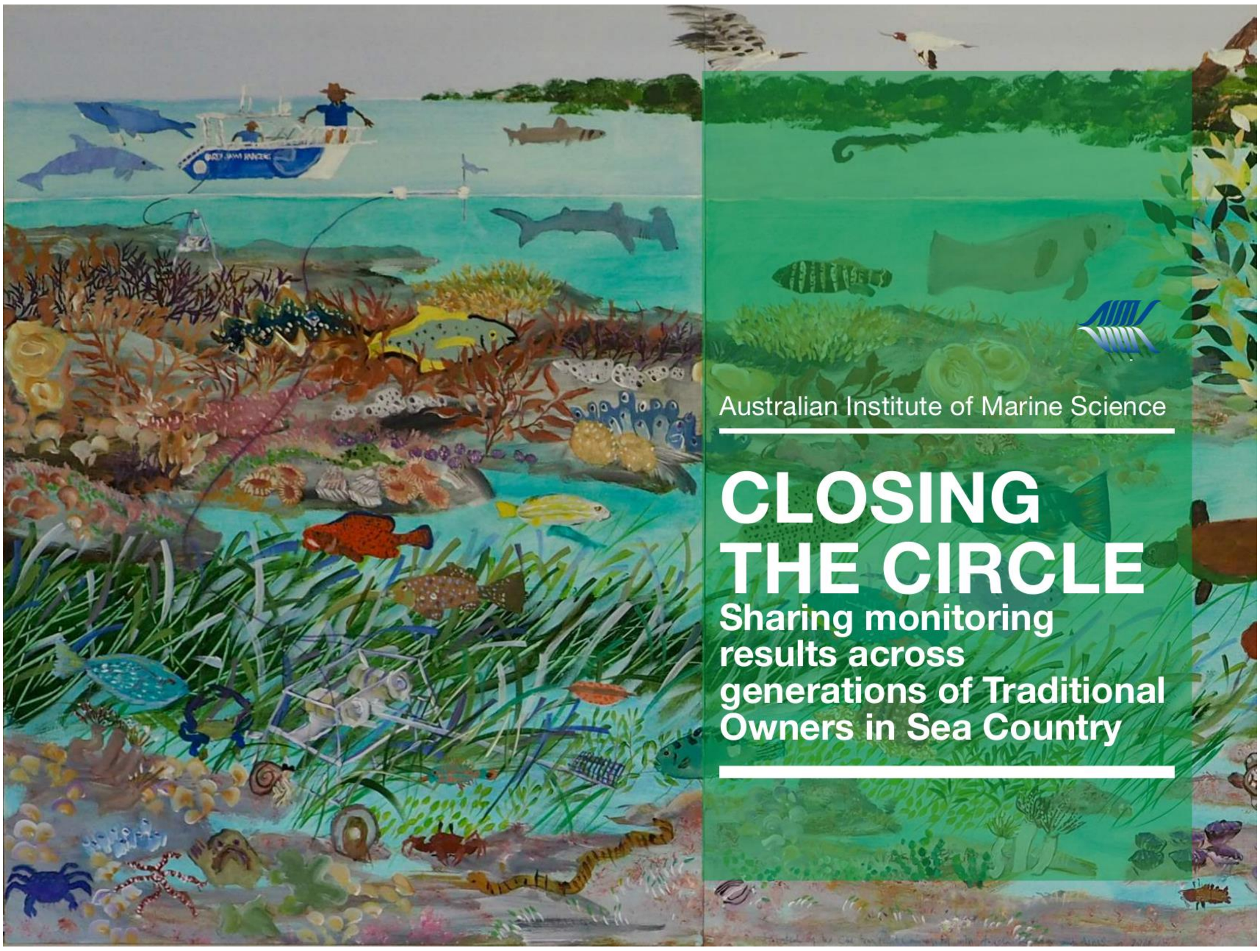

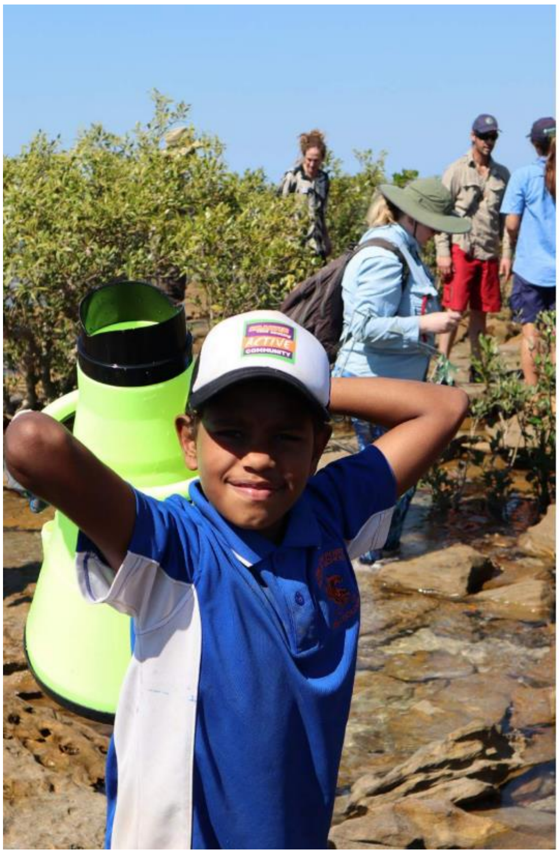

On-country workshop

May 10-14, 2021, One Arm Point, Kimberley, WA

The Australian Institute of Marine Science together with the Bardi Jawi and Oorany Rangers, ran a workshop to evaluate methods of effectively delivering marine monitoring results across multiple generations of Traditional Owners. AIMS and the Bardi Jawi Rangers have a sea country monitoring partnership since 2018, following trends in fish, benthos and temperature.

Participants included AIMS (Katherine Cure, Jim Underwood, Martial Depczynski), Bardi Jawi and Oorany Rangers, One Arm Point Remote Community School, Bardi Jawi Ranger Steering Committee, the University of Western Australia (Jane Prince, Matilda Murley), Angela Rossen (artist and biodiversity educator), and Sam Frederick (film maker).

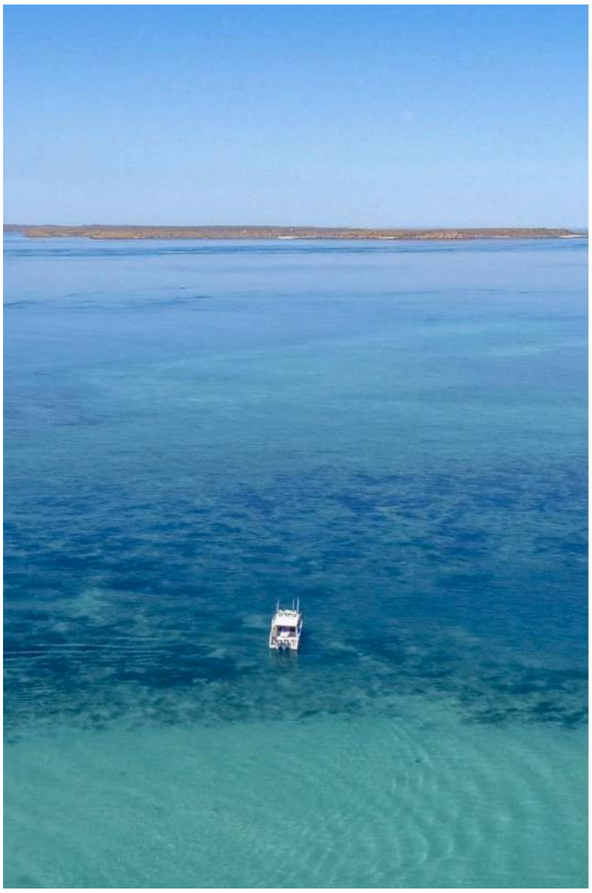

"If our country is healthy, then our people are healthy too, and we want it to stay that way. AIMS and the Bardi Rangers are working together checking and monitoring the health of our reef, sea weeds, corals, sponges and other marine life. The week AIMS spent in Bardi One Arm Point, was to update the community on the project that has been going on for three years and getting the school children involved; this is a great project."

- Kevin George, Senior Cultural Manager, Bardi Jawi Rangers

One Arm Point Remote Community School

Whole School Field trip

- Exploration of marine life (cameras, bathyscopes)
- ArcGIS Field Maps App
- Counting marine life inside quadrats
- Drawing marine/coastal life
- Invertebrate collections and display in trays
- iSCOPE magnification of small marine life

Small Group Activities

- Video making as a report
- Sea Country art (drawing and painting mural)
- Data collection, annotation and basic plotting (transects, quadrats, BRUVS, barplots)
- Guess Who? Sticky notes on forehead game
- Large map and post-its activity to share fishing/ camping spots, tides, traditional names, etc.

4

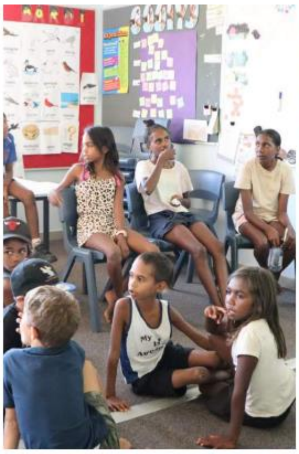

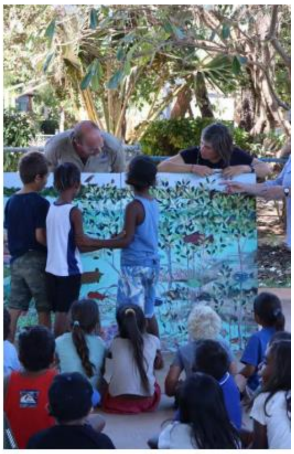

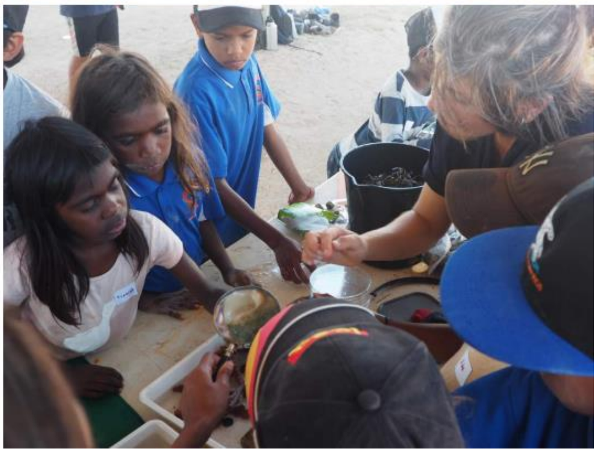

Bardi Jawi Ranger Base

Sharing monitoring data with rangers and Elders

1. Meetings after school hours for AIMS scientists to present results
2. Discussion of communication materials, meaning, and suitability
3. Co-creation of presentation to Ranger Steering Committee and Elders
4. Ranger led presentation to Steering committee and Elders- evaluation of use of communication materials

5

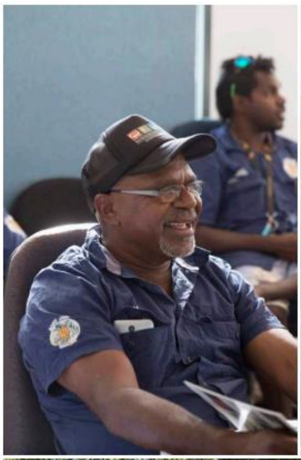

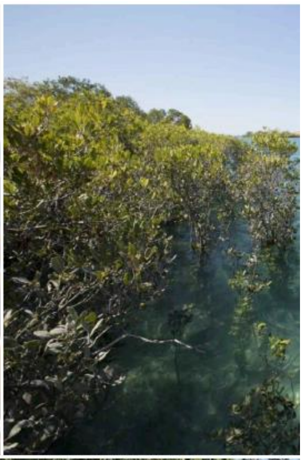

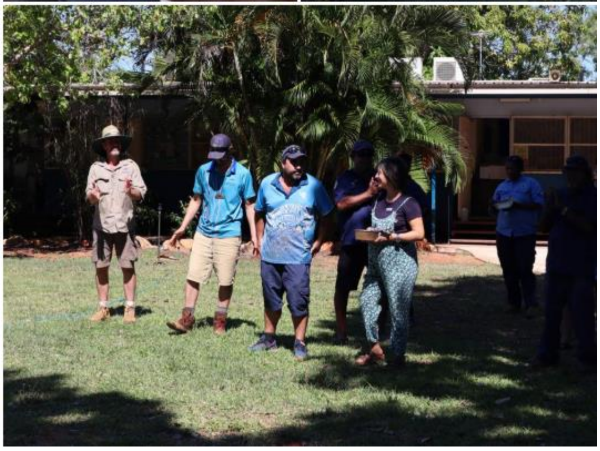

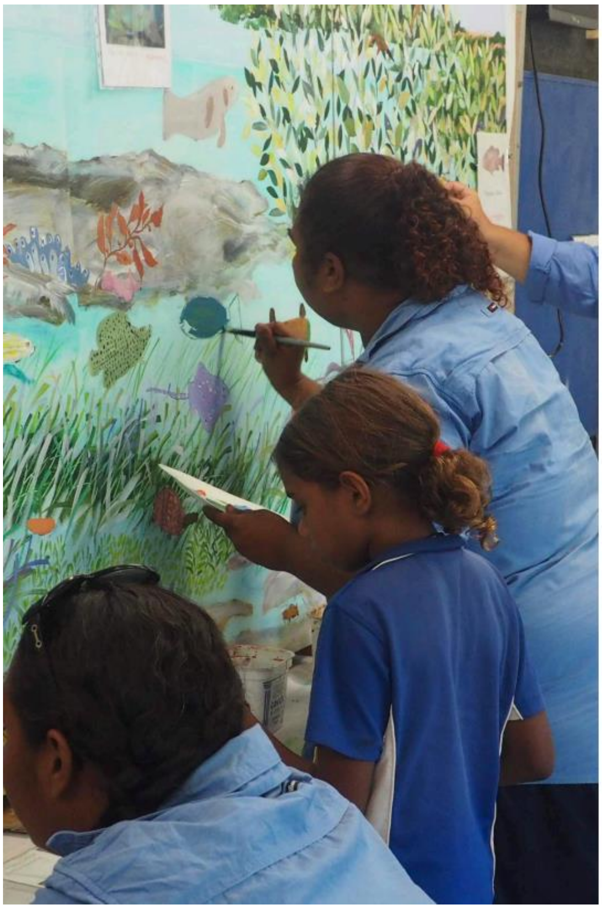

Products Trialled

The following tables show six different communication products trialled during the week's workshop in various settings and with several audiences. Pros and cons for each are discussed.

STORY MAP

online scroll through presentation, ArcGIS

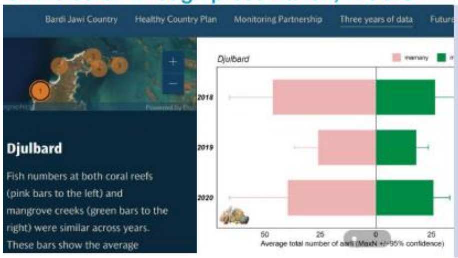

Target Audience	PROS	CONS	Feedback
Rangers	Appealing graphics	Requires high speed internet to view and make	•Used as a reporting tool •Better self explanation of graphs needed for users not familiar with axes, labels, colour, etc •Needs to load faster •Looks great when it works •Can be used as a powerpoint and preferred to latter as presentation style (more dynamic and visually attractive)
General Public	Scrolls like a presentation; can be self-guided or directed at audience	Customizable to an extent; lack of control of visuals	
	Can include diverse media types (e.g. audio, video, graphs)	Private sharing requires GIS license	
	Simple style for summary and reporting	GIS license required to make and expensive	
	Can use as backbone and update graphs annually with new results		

REPORT CARD

Printed A3 flip book brochure

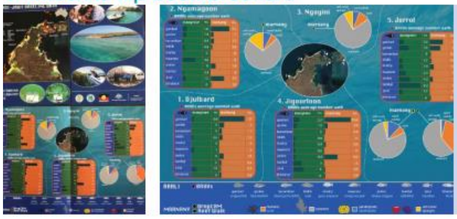

Target Audience	PROS	CONS	Feedback
Rangers	Printed; can sit and yarn	Physical distribution needed	•A format useful for informal yarns with a cup of tea •Graphs need to be simple enough for all to understand without many words. This is tricky, unless just a very simple traffic light system with coloured dots •Best for aid in personal explanations to clients/ audience
General Public	Simple and short- to the point	Time consuming to produce well as not automated	
	Great for distribution in several sizes	Requires graphic design skills	
	Effective summary	Doesn't include details about project, statistics, etc.	

POWERPOINT

Microsoft presentation product

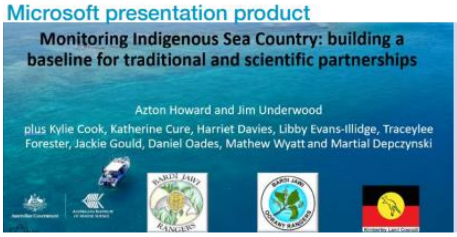

Target Audience	PROS	CONS	Feedback
Rangers	Familiar	Can be boring, long and static	•The most widespread use and most familiarity for all users •Used by rangers and scientists alike •Versatile and can be used in a more discussion type format if the audience is involved and more of a conversation
Schools	Easy to create, use and share	Typically delivered by a presenter in front of an audience, causing disconnect	
Elders			
Steering Committee	No internet required	Requires good presenter	
General Public	Customizable for any audience		

VIDEO
Professional, amateur, BRUVS reels

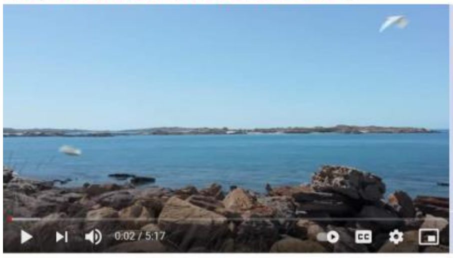

Target Audience	PROS	CONS	Feedback
General Public<br>*Community all ages*	Shared and viewed many times; loved by all	Expensive to produce if professional	•Preferred product to share<br>•Great aid for shy presenters<br>•Quickly available when needed<br>•Can be embedded into other presentations<br>•Continued use<br>•Used by school kids to report on activities; a big highlight<br>•BRUVS reels are a favourite across age groups; good way to connect and share language names
	Great for story telling, and for feeling a connection to people and country	Hard to show data with; more of a story	
	Can be used across generations and literacy levels		
	Can be shared via internet, but also saved and viewed offline		
	Personal and beautiful		

POSTER
Large printed rollout, sturdy board

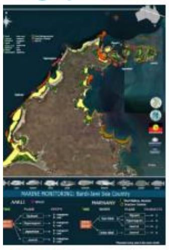

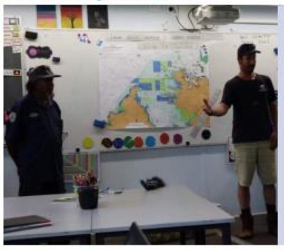

Target Audience	PROS	CONS	Feedback
General Public<br>*Rangers*	Large and interactive	Can be time consuming to design and create	•Stiff aluminium format worked best for activities and pointing to<br>•Good to find space for constant exposure in community so not forgotten; cultural centre, school<br>•Design based on map<br>•Use minimal information; if graphs, they need to be simple and self-explanatory for posting in community
	Perfect for activities with audience; ex: sticking post-its to highlight location based attributes	Requires some graphic skills and access to good quality printing	
	Map based and easy for Aboriginal Australians to relate to	Can be stashed away and forgotten in the office	
	Can be arranged on table/floor getting rid of front of audience figure		

MURAL
Large canvas; 3 MDF boards 90*120 cm

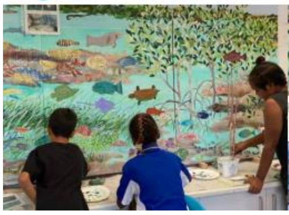

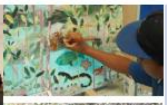

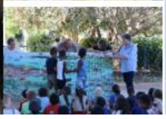

Target Audience	PROS	CONS	Feedback
General Public<br>*Children*	Permanent exhibit	Time consuming on-site	•All children loved participating; a highlight even for 12+ year olds and rangers<br>•Fantastic sharing experience; a glue to the week's workshop<br>•Limitations in interaction with children about monitoring and science while drawing/ painting, unless artist can relay/share the message
	Good across all age groups, even when children targeted	Limited to showcasing biodiversity; not good for data	
	Large and visually appealing; emotive as a compilation		
	Information without words		
	Self explanatory for community		

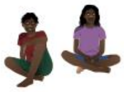

Children ~12 years old understand the concept and how to generate simple bar graphs of means

Break it up with games, activities

Students loved making videos to present findings

Partner with classroom teachers in advance if working at school

Rangers need to merge and interpret science within a cultural framework including community expectations and traditional law and lore

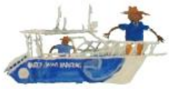

Rangers took on comms materials provided and used them effectively to share results of monitoring with Elders/ Steering

Rangers can deal with more complex science outputs than Elders or children

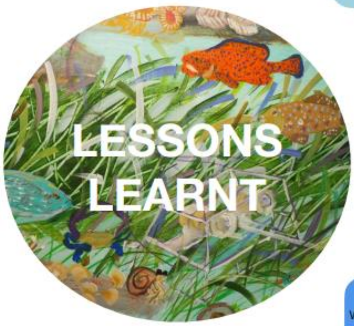

Remote communities are filled with uncertainty. It is difficult to plan from off-site. Partner with known local champion and be flexible!

Anything to do with fish is a winner across age groups and genders

Know your audience and discuss report outputs/types/ formats in advance with partners

Art (painting) is a powerful and connecting medium across generations for science transfer

Small groups for focused interactions work best

Line graphs connecting means through time were the most easily understood

Elders requested printed small brochures with information to take with them when visiting on-country

Diverse communication materials are needed including print, graphs, videos

Multi-gender teams that can speak/reach out to boys/men and girls/women separately are ideal

Flexibility is key; make plans on the go; prepare list of options and activities; feel and adapt; work with the team

Elders get western science; most can understand simple graphs and figures showing trends through time.

ALL

Make graphs as simple as possible so they are self-explanatory; infographic style is best

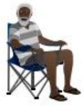

9

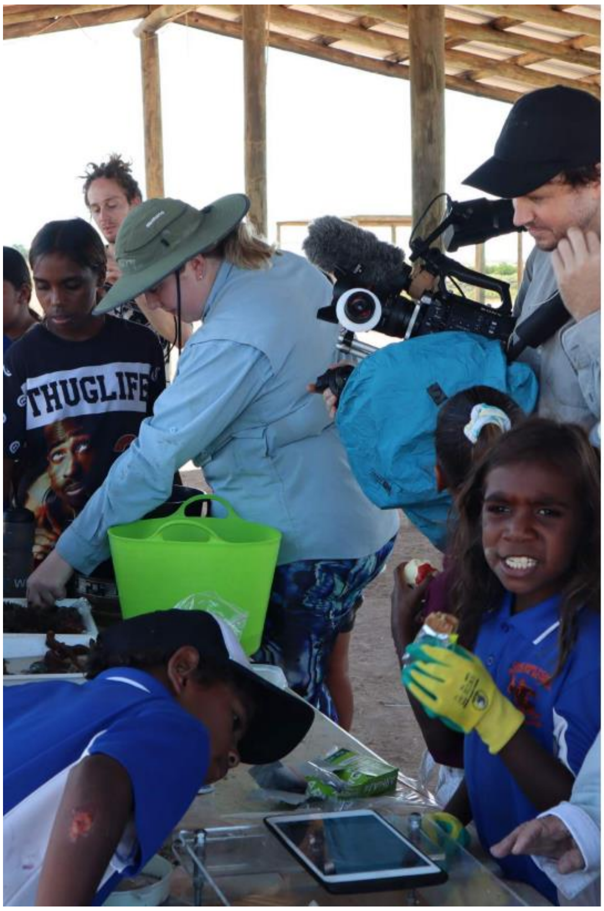

AIMS researchers thank the Traditional Owners of Bardi Jawi country for their ongoing commitment to keeping a healthy sea country, learning two way with western scientists, welcoming us and keeping us safe in their land and sea. This project was funded by an AIMS Capability Development Fund, the Western Australian Marine Science Institution and Inspiring Australia. Symbols courtesy of the NESP Northern Australia Hub, nespnorthern.edu.au.

For more information contact *Katherine Cure* (k.cure@aims.gov.au)

Australian Government | Australian Institute of Marine Science
